# Sensing Spin Precession with Free Electrons

**DOI:** 10.1021/acsnano.5c13351

**Published:** 2026-01-20

**Authors:** Antonín Jaroš, Michael S. Seifner, Johann Toyfl, Benjamin Czasch, Santiago Beltrán-Romero, Isobel C. Bicket, Philipp Haslinger

**Affiliations:** Vienna Center for Quantum Science and Technology, Atominstitut, USTEM, 27259 Technische Universität Wien, Stadionallee 2, Vienna 1020, Austria

**Keywords:** spin, electron spin resonance, electron paramagnetic resonance, spin precession, microwave spectroscopy, free electron, transmission electron microscopy

## Abstract

We present a method that combines spin resonance spectroscopy with transmission electron microscopy (TEM), enabling localized *in situ* detection of microwave (MW)-driven spin transitions in the specimen, utilizing the free-space electron beam of the TEM as a signal receiver. Spin state polarization is achieved via the magnetic field of the TEM’s polepiece, while a custom-designed microresonator integrated into a TEM sample holder delivers continuous wave MW excitation at GHz frequencies. At resonance, the MW field drives spin transitions that produce a dynamic, precessional magnetic field around the specimen, inducing a deflection of the free-space electron beam. Phase-locked detection synchronized to the MW driving fields enables the isolation of spin precession contributions to the electron beam deflection. The presented technique offers a pathway for the *in situ* exploration of spin excitations at the nanoscale.

## Graphical abstract

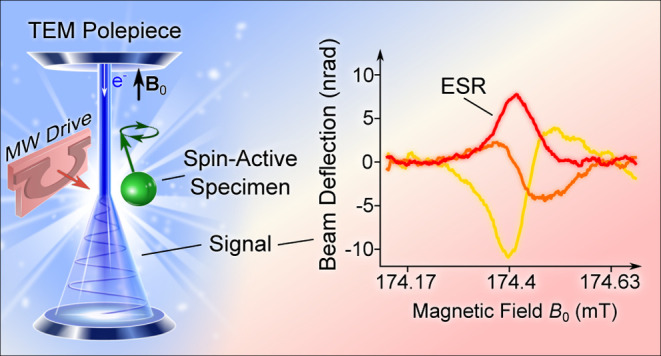

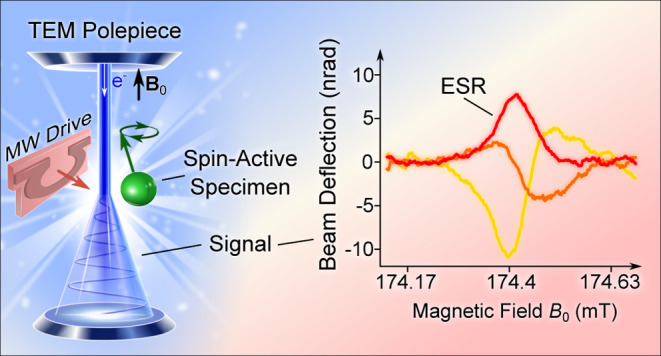

## Introduction

The microwave (MW) frequency band plays a crucial role in many scientific fields. In atomic and molecular quantum optics, it enables the coherent manipulation of long-lived transitions, such as the cesium hyperfine transition which defines the second.^1–4^ MW spectroscopy has also proved disruptive in other disciplines such as condensed matter physics, chemistry, biology, and medicine, where electron spin resonance (ESR),^5,6^ nuclear magnetic resonance (NMR)^7,8^ and ferromagnetic resonance (FMR)^9,10^ are key to advancements in noninvasive quantum technologies, including magnetic resonance imaging (MRI).^11^

Such technologies rely on sensing a fundamental quantum property of matter: the spin. MW radiation is used to coherently manipulate spin states, which exhibit surprisingly long coherence times.^7,12,13^ The chemical environment can alter these spin states, providing spectroscopic insights into the atomic structure of a given sample. However, conventional spectroscopic tools typically average over macroscopic samples and struggle to probe spatial variances within the specimen. Enhancing both the sensitivity and spatial resolution of spin-detection techniques is therefore crucial for advancing the study of spin ensembles down to the atomic scale.

To date, extensive efforts have been made to detect spin excitations through diverse mechanisms, including Brillouin light scattering (BLS),^14–17^ electrically detected spin-torque FMR (ST-FMR),^18–21^ optically detected magnetic resonance (ODMR),^19,22,23^ scanning tunneling microscopy (STM)-based techniques,^24–27^ and experiments hosted at large-scale synchrotron facilities,^28–30^ among the many other possibilities.^31–35^ Yet, spin detection at the nano- and atomic-scale remains highly constrained, typically limited to only specific sample geometries and instruments.^23^ The possibility to use free electrons, which respond to local magnetic field variations such as those induced by spin-state changes,^36^ opens a more accessible pathway for nanoscale spin detection.^37^

In particular, transmission electron microscopy (TEM) is a powerful technique that enables atomic-scale investigations using a highly controlled free-space electron beam.^38–41^ Ultrafast TEMs with laser-triggered sources^42–47^ or chopped electron beams^48,49^ extend those capabilities by probing dynamic processes on femto- and even atto-second time scales. For detailed spectroscopic specimen analysis, TEM offers an advanced suite of analytical techniques such as electron energy loss spectroscopy (EELS).^50^ Recent advancements allow for the detection of energy losses and gains associated with optical interactions,^45,51,52^ atomic vibrations (i.e., phonons) and mid-IR plasmonic excitations,^53,54^ and, more recently, THz spin excitations.^55^ Nevertheless, the MW frequency band (∼ μeV) is not accessible in EELS due to the energy width of the primary electron beam. Consequently, new techniques need to be developed to study phenomena in this frequency regime.

Recent research efforts focus on the development of custom-made sample holders capable of exciting samples at MW frequencies.^6,56–59^ Stroboscopic MW-pump electron-probe schemes were utilized, enabling, e.g., ultrafast imaging of magnetic vortex cores,^57,58,60^ probing of beam deflections near MW circuits,^59,60^ and the visualization of spin waves in ferromagnetic thin films.^61^ In prior work, we integrated a specially designed MW resonator on a custom TEM sample holder, allowing for conventional ESR (c-ESR) investigation of miniaturized sample sizes within the TEM environment.^6^ This setup complements commercially available TEM equipment by realizing coherent spin manipulation at frequencies around 4.89 GHz, corresponding to an excitation energy of ≈ 20 μeV.

Using the excitation field produced by this setup, we drive specimen spin states with MWs and read out the response using the free-space electron probe positioned near the specimen in aloof mode (in which the probe is located in vacuum just outside the specimen), effectively acting as a localized receiver. The electrons experience deflection due to the Lorentz force: **F** = −*e* **v** × **B**_dyn_, where **B**_dyn_(**r**, ω, *t*) is the dynamic magnetic field generated at position **r** by the specimen’s magnetization precession. Since the electron beam is also modulated by the continuous-wave (CW) MW excitation field, the detection is phase-locked: both the specimen’s magnetization precession and the induced electron beam deflection are synchronized with the driving MW field. This approach allows us to isolate and study precession-induced intensity variations visible in the electron beam’s angular distribution with picoradian (prad) sensitivity, while picosecond (ps) temporal dynamics are encoded in the beam’s momentum and phase-averaged over many MW cycles. The readout with the free-space electron probe, therefore, does not require the modulation fields or lock-in detection schemes used by traditional ESR spectrometers.

This developed CW MW-pump electron-probe scheme enables the direct investigation of spin signatures imprinted in the electron beam, which we refer to as SPINEM (SPIN Electron Microscopy), drawing a parallel to the established technique of photon-induced near-field electron microscopy (PINEM).^45,62^ Building on the capabilities of modern TEM,^43,63,64^ further improvements of the presented technique could enable investigations of coherently driven spin phenomena at the atomic scale.

## Results and Discussion

### Theoretical Background of SPINEM

To create the driving field for the SPINEM measurements, we combine the input electronics for a c-ESR setup, similar to those in refs 8,65,66 with a custom-built TEM sample holder.^6^ The holder features an Ω-shaped microresonator, impedance-matched to 4.89 GHz. The electron spin-active specimen (α,γ-bisdiphenylene-β-phenylallyl, BDPA^67^) is positioned near the center of the microresonator, while ensuring sufficient space to perform aloof electron-probe measurements. We operate the TEM in low-angle diffraction (LAD) mode, using the objective lens (OL) to produce a **B**_0_ field in the range of 174 mT at the specimen, which is used both for spin polarization and for tuning the resonance condition by adjusting the specimen’s Larmor precession frequency to match the optimal 4.89 GHz impedance of the microresonator. Without MW driving fields applied, the magnetization vector of the spin states **M** aligns with the static magnetic field **B**_0_ = (0, 0, *B*_0_), which is oriented along the TEM’s optical axis. Consequently, no deflection of the electron probe is observed, as shown in Figure 1(a)­(i).

**Figure 1:**
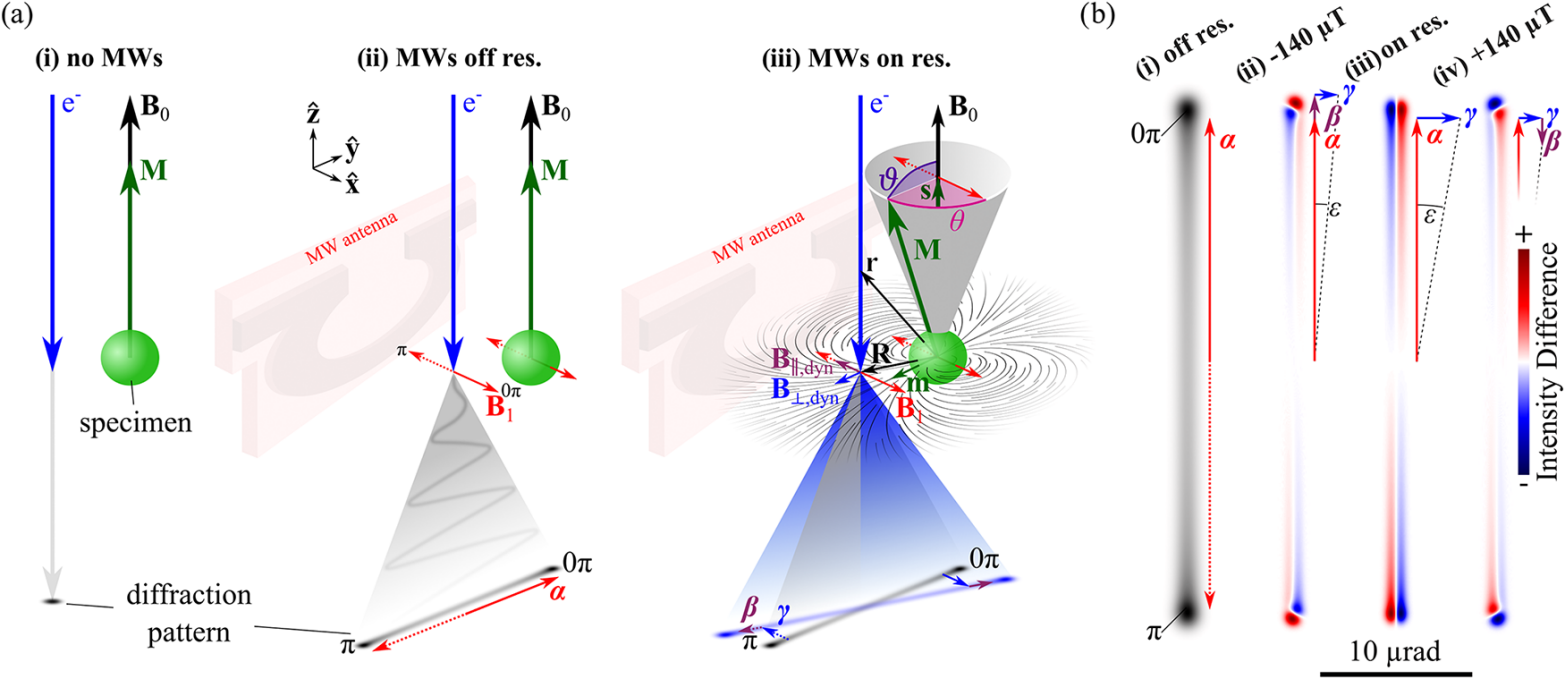
CW SPINEM *in situ* experiment in a TEM. (a) A specimen containing addressable spin states is positioned within the static magnetic field **B**_0_ of the TEM objective lens polepiece. In the absence of a MW driving field (i), the specimen magnetization **M** aligns with **B**_0_, resulting in minimal interaction with the free-space electron probe and, consequently, no observable angular beam deflection at the camera plane. When MWs are applied at frequencies far from resonance (ii), the dynamic magnetic field **B**_1_ induces an electron beam deflection via the Lorentz force, with magnitude |**α**(ω, *t*)| ∼ ∫|**B**_1_(*z*, ω, *t*)|, while the specimen magnetization remains unperturbed. At resonance (iii), the spin states are driven by the **B**_1_ field, causing **M** to precess at an angle ϑ around **B**_0_, lagging by a phase θ relative to the driving field. This precession gives rise to a static out-of-plane component **s**(ω) and a dynamic in-plane component **m**(ω, *t*), such that **M**(ω, *t*) = **s**(ω) + **m**(ω, *t*). Additional beam deflections appear both perpendicular (|**β**(**R**, ω, *t*)| ∼ ∫|**B**_∥,dyn_(**r**, ω, *t*)|) and parallel (|**γ**(**R**, ω, *t*)| ∼ ∫|**B**_⊥,dyn_(**r**, ω, *t*)|) to the primary deflection direction defined by **α**. These arise from the dynamic fields **B**_∥,dyn_ and **B**_⊥,dyn_ generated by **m**(ω, *t*) at the electron probe position **r** = **R** + (0, 0, *z*), with **R** = (*x*, *y*, 0). (b) Simulated electron beam profiles on the camera. Off resonance (i), the beam profile corresponds to the time-averaged projection of the sinusoidal beam deflection |**α**(ω, *t*)| ∼ cos­(ω*t*), where ω*t* denotes the MW phase. Near resonance, the evolving magnetization **m**(ω, *t*) distorts the deflection pattern, resulting in its stretching, compression, and tilt by an angle ε. These effects are visualized in difference images (ii–iv), obtained by subtracting the reference image in (i) from the calculated patterns at the indicated magnetic fields relative to the resonance condition. The difference images highlight the dynamic coupling between the MW-driven spin motion and the transmitted electrons. Further details of the calculations are provided in the Methods section.

Irradiating the sample with a time-dependent magnetic field **B**_1_(*t*) = (*B*_1_(*t*), 0, 0), **B**_1_ ⊥ **B**_0_, generated by the microresonator induces dynamic spin evolution. The coherent interaction between **B**_1_(*t*) and the sample’s spin states causes a precession of the spins and the corresponding magnetization, **M**(ω, *t*). The magnetization follows the Bloch equations,^7^ which capture the characteristic dynamics of ESR spectroscopy. Solving these equations yields the resonance condition ω_res_ = 2πΓ*B*_0_, where Γ ∼ 28 GHz/T is the gyromagnetic ratio. The resonance condition is established by sweeping the microwave frequency or static field *B*_0_, during which the magnetization varies characteristically, reflecting intrinsic properties of the specimen.

When the applied CW field is off-resonant, the magnetization vector **M** remains parallel to **B**_0_, while the electron beam experiences a total angular deflection **α**(ω, *t*) due to the dynamic Lorentz force, integrated across the electron’s trajectory, as illustrated in Figure 1(a)­(ii)

1 $$\alpha \left(\omega ,t\right)∼\int_{-\infty }^{+\infty }v_{e}\times B_{1}\left(z,\omega ,t\right)dz$$

where **v**_e_ = (0, 0, −v_e_) is the velocity vector of the electron beam and the *z*-axis aligns with the optical axis. As the microresonator spans a large portion of the accessible sample region, the **B**_1_(*z*, ω, *t*) amplitude exhibits only minor variations across the *xy*-specimen plane,^6^ which we neglect here. Furthermore, we employ a quasi-static approximation in this context. A single MW cycle takes around 1/4.89 GHz^–1^ ≈ 204 ps, whereas the electron interacts with the microresonator fields for $\frac{L}{v_{e}}\approx 5\,\;\mathrm{ps}$ , assuming an interaction length of *L* = 1 mm. This time scale disparity justifies the integration over the spatial coordinate, rather than over time. For details on the calculations of **α**, see Supporting Information (SI) A.

As a result, the electron beam is sinusoidally modulated, **α**(ω, *t*) = **α**_max_(ω) cos­(ω*t*). With a conventional TEM, we are not able to temporally resolve the 204 ps period of the oscillating electron probe. On the camera, the resulting electron deflection pattern represents a time-averaged projection of the sinusoidally modulated electron beam, with length 2α_max_. For a camera exposure time of 5 s, the final image represents a sum of ≈2.4 × 10^10^ MW cycles. However, different points along the pattern correspond to different *ωt* phases of the driving field, effectively creating a position-encoded time resolution, similar to ref 68. Figure 1(b)­(i) shows a calculated example of such a pattern, and Figure S1­(a) represents the line profile along the pattern. The pattern length varies with both the *B*_0_-field sweep, through small magnification changes from the objective lens (Figure S1­(b)), and the MW frequency sweep. The latter dependence dominates, as **α**_max_(ω) scales more strongly with **B**_1,max_(ω), reflecting the microresonator’s impedance match and MW power delivery to the location of the electron probe (Figure S2).

For CW MW excitation at and near the resonance, **B**_1_(ω ≈ ω_res_), the resulting deflection pattern is imprinted with the phase-locked spin signature—additional beam deflections both parallel, **β** (**R**, ω, *t*), and perpendicular, **γ** (**R**, ω, *t*), to **α**(ω, *t*) arise from the dynamic in-plane component **m**(ω, *t*) of the precessing magnetization **M**(ω, *t*) = **m**(ω, *t*) + **s**(ω), where **s**(ω) is a static component aligned with **B**_0_ that does not contribute to the deflection. Here, **R** = (*x*, *y*, 0) represents the position of the electron probe relative to the specimen, in the specimen plane. Considering an interaction of less than 5 ps, the magnetization precession dynamics (with a 204 ps period) can be considered effectively static. A theoretical derivation of **β** and **γ** deflections is presented in SI B. The deflections can be expressed by

2 $$\beta \left(R,\omega ,t\right)∼\int_{-\infty }^{+\infty }v_{e}\times B_{∥,\mathrm{dyn}}\left(r,\omega ,t\right)dz$$

3 $$\gamma \left(R,\omega ,t\right)∼\int_{-\infty }^{+\infty }v_{e}\times B_{⊥,\mathrm{dyn}}\left(r,\omega ,t\right)dz$$

where **B**_∥,dyn_(**r**, ω, *t*) and **B**_⊥,dyn_(**r**, ω, *t*) denote, respectively, the collinear and perpendicular in-plane components of the **B**_dyn_(**r**, ω, *t*) = **B**_∥,dyn_ + **B**_⊥,dyn_ + **B**_*z*,dyn_ dynamic magnetic field relative to the driving field **B**_1_(*z*, ω, *t*) at the electron probe position **r** = (*x*, *y*, *z*), as illustrated in Figure 1(a)­(iii). The electron probe is not sensitive to the out-of-plane **B**_*z*,dyn_(**r**, ω, *t*) component. Thus, **β**(**R**, ω, *t*) and **γ**(**R**, ω, *t*) define a measurement scheme that not only yields the amplitude of the dynamic magnetic field integrated along the electron’s trajectory, but also enables the evaluation of the in-plane field vector’s direction. This is analogous to conventional scanning TEM (STEM) techniques,^69,70^ such as differential phase contrast (DPC) or 4D-STEM, which enable mapping of static or slowly varying electric and magnetic fields at the atomic-scale.^71–73^ In contrast, SPINEM probes phase-locked dynamic magnetic fields at GHz frequencies. The influence of electric fields from the microresonator or the spin system is minor, as discussed in SI C and Figure S3, and is therefore neglected in eqs 1–3

The dynamic field **B**_dyn_(**r**, ω, *t*) is generated by the specimen’s in-plane magnetization **m**(ω, *t*). Consequently, both quantities vary with detuning from resonance as the magnetization **M**(ω, *t*) precesses at an angle ϑ­(ω) relative to the static spin alignment **s**(ω), while lagging behind the driving field phase *ωt* by a phase shift θ­(ω). Owing to these effects, it can be shown that, for a sweep across the resonance, **β**(**R**, ω, *t*) and **γ**(**R**, ω, *t*) also each correspond to either an absorption or dispersion spectrum of c-ESR or a linear combination thereof, see SI B for a derivation.

Although we do not have the time resolution required to directly resolve a single precession, the phase-encoded deflections **β**(**R**, ω, *t*) and **γ**(**R**, ω, *t*) result in an electron redistribution, which we can observe via subtraction of the image from a far off-resonant reference beam image, shown in Figure 1(b)­(i). Calculated difference images at a specific electron probe position **R**, visualized in Figure 1(b)­(ii–iv), illustrate the effect of **β** and **γ**. The deflection **β** results in an expansion of the electron pattern at a *B*_0_ detuning of −140 μT, and a compression at +140 μT from resonance. Simultaneously, the pattern tilts by an angle ε around the optical axis as a result of **γ**, with the largest pattern tilt occurring at resonance. Consequently, evaluation of the pattern’s longitudinal spread, α_max_(ω) + β_max_(**R**, ω), and tilt, ε­(**R**, ω)—relative to the off-resonance reference pattern and resulting from the combined effect of **γ**(**R**, ω, *t*) and **β**(**R**, ω, *t*)—enables quantitative measurement of the time-independent spin-induced signal, as described in SI B.1 and B.2. From these quantities, we can determine γ_max_(**R**, ω) and β_max_(**R**, ω), the maximum deflection components at the pattern extrema

$$\gamma_{\max}\left(R,\omega \right)=\alpha_{\max}(\omega )\;\;\tan\varepsilon \left(R,\omega \right)$$ 4

Under the calculation conditions used in Figure 1(b) (**B**_1_ amplitude and frequency, **B**_0_ strength, **R**, *T*, etc.), chosen to match the experimental values, β_max_(**R**, ω ≈ ω_res_) and γ_max_(**R**, ω ≈ ω_res_) are expected to reach a maximum amplitude of only ∼10 nrad. This is several orders of magnitude below the beam deflections typically observed in state-of-the-art 4D-STEM measurements, which can reach angular sensitivities of ∼1 μrad.^74,75^ For comparison, low-order Bragg diffraction results in beam deflections on the order of 10 mrad.^76,77^

### SPINEM Experiments

Figure 2 illustrates the results of our experimental SPINEM measurements, employing a CW MW-pump and electron-probe scheme. The electron probe is placed near the edge of the specimen in aloof mode. The OL excitation is varied to tune the *B*_0_ field while keeping the MW excitation frequency constant, performing a magnetic field scan over the resonance. We avoid sweeping the MW frequency in order to remain at the optimal impedance match of the microresonator, ensuring a stable and linearly polarized **B**_1_ field. Further details on the experimental parameters can be found in the Methods section.

**Figure 2:**
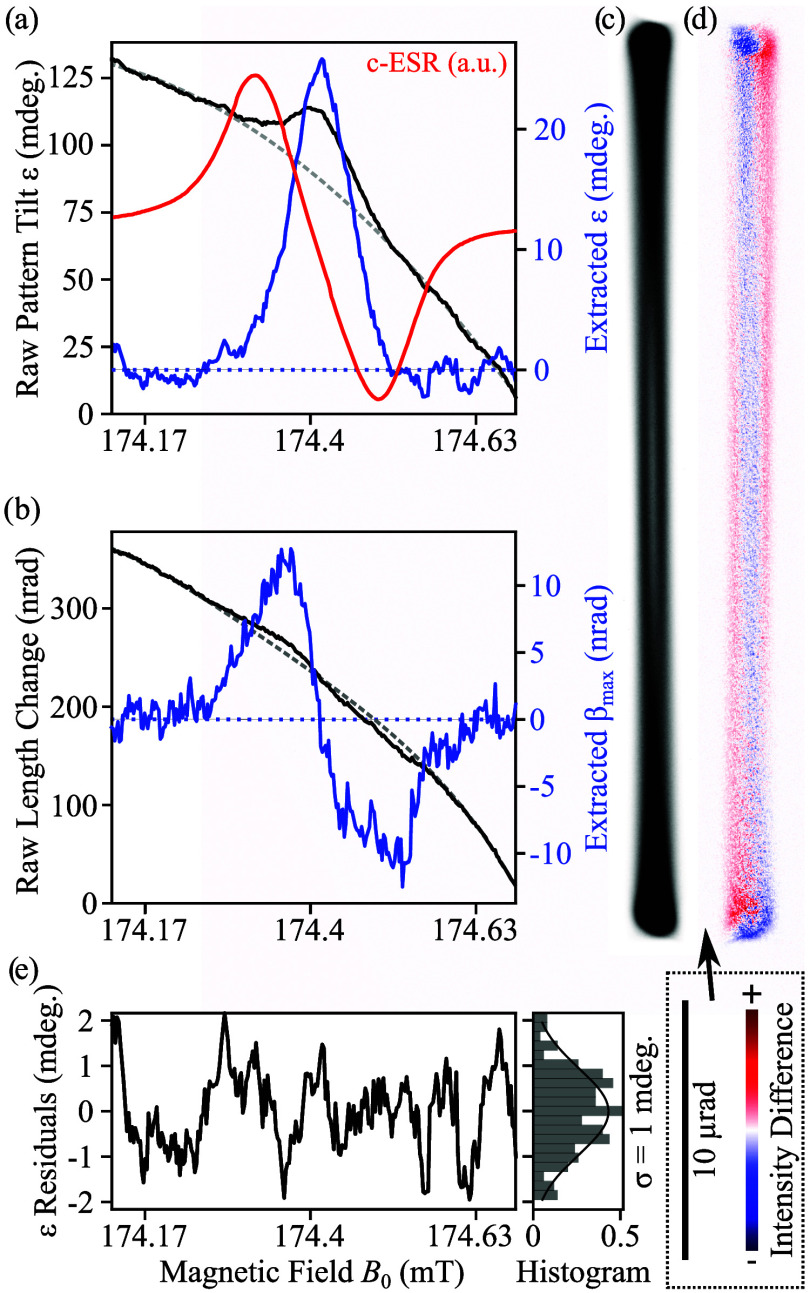
CW SPINEM measurement. (a) Pattern tilt ε­(ω) resembles an ESR absorption spectrum measurement. (b) Pattern length change corresponds to the detection of an ESR dispersion spectra. For these two plots, measured data is shown in black, the fitted polynomial offset is displayed in dashed gray, and the final signal in blue. For reference, a c-ESR absorption spectrum measurement is shown in (a) (red). The far-off resonance image (c) is subtracted from the pattern on-resonance to reveal the difference image (d), which highlights the tilting ε at resonance, with positive and negative intensity differences shown in red and blue, respectively. (e) Fit residuals of pattern tilt ε, following a Gaussian distribution with a standard deviation of 1 millidegrees (mdeg.). This corresponds to an SNR of 26 for the measurement in (a). c-ESR experimental conditions: MW output power *P*_g_ = 24 dBm, lock-in time constant τ = 30 ms, modulation frequency 101.01 kHz. SPINEM experimental conditions: MW output power *P*_g_ = 24 dBm, camera acquisition time 20 s per spectral point, beam current 3.9 pA.

Following the data processing steps also detailed in the Methods section, Figure 2(a) presents the extracted SPINEM pattern tilt ε (black curve). Varying the OL excitation induces a background tilt, which is accounted for by fitting and subtracting an offset (dashed curve). The resulting signal (blue curve) is similar to c-ESR absorption spectra. At resonance, we extract a maximum pattern tilt of ε ≈ 26 millidegrees (mdeg.), caused by the dynamic spin precession. The change of the pattern length, proportional to the β_max_ parameter (maximum of β), is shown in Figure 2(b), revealing strong similarities to c-ESR dispersion spectra. We observe a maximum pattern elongation of approximately 12 nrad. The background corresponds to the change of image magnification caused by the OL sweep (see Figure S1­(b) for details).

For reference, we measured an *in situ* lock-in c-ESR spectrum (in red) of the same specimen immediately following the SPINEM measurement. Note that lock-in detection of the c-ESR captures the derivative of the absorption signal, producing a profile visually similar to ESR dispersion spectra. Our SPINEM measurement matches well with the c-ESR reference in both resonance frequency and line width. The c-ESR measurement exhibits a full width at half-maximum (fwhm) of 5.6 ± 0.1 MHz due to broadening caused by the field modulation required for obtaining c-ESR measurements via the lock-in detection scheme.^6^ In contrast, the SPINEM absorption spectrum (Figure 2(a)) does not require field modulation, and reaches a fwhm of 3.1 ± 0.2 MHz.

Electron beam patterns are captured at a nominal camera length of 600 m in LAD mode, reaching a maximum deflection α_max_ of 16.65 μrad in the example of the far off-resonance case shown in Figure 2(c). The difference image in Figure 2(d), obtained by subtracting the off-resonant pattern from the pattern at resonance, visualizes the pattern tilt ε at resonance. A tilt of 26 mdeg. results in an electron beam displacement of 0.7 pixels at the pattern end point or, equivalently, an angular deflection amplitude γ_max_ = α_max_ tan ε of 7.5 nrad (eq 4). This deflection is a direct consequence of the spin-electron interaction.

For our measurements, we optimize the MW power to maximize SPINEM sensitivity, as quantified by the signal-to-noise ratio (SNR). For a comparison of SNR and MW power, see SI Figure S4; generally, increasing the MW power increases the length of the electron beam pattern and improves the sensitivity of the measurement. Figure 2(e) presents the residuals from the fit of pattern tilt *ε*, which reveals a standard deviation of ∼1 mdeg., corresponding to a γ_max_ deflection uncertainty of ∼290 prad and a beam displacement sensitivity of ∼1/37 of a pixel on the Gatan Rio 4K camera. The long camera length in LAD mode and the phase-locked nature of γ and β deflections enable this angular sensitivity, which results in an SNR of 26 and a spin sensitivity of *N*_min_ ≈ 5.6 × 10^14^ spins/ $\sqrt{\mathrm{Hz}}$ . Although the sensitivity of the SPINEM method in our proof-of-principle measurements remains several orders of magnitude lower than that of benchmark c-ESR, the scaling of *N*_min_ with several key experimental parameters—as elaborated on in SI D (Spin Sensitivity Estimates)—indicates substantial potential for future improvement: magnetic field (∝ 1/*B*_0_), temperature (∝ *T*), beam current ( $\propto 1/\sqrt{I_{e}}$ ), electron velocity (∝ *v*_e_), and the specimen-probe distance (∝ |**R**|^2^). Specifically, employing a focused STEM probe can enhance both the spatial resolution of SPINEM as well as the detectable signal by several orders of magnitude. Figure S5 demonstrates the retrieval of the pattern tilt using a focused low-magnification (LM)-STEM probe. The convergence of the STEM probe results in a disk in momentum space and a change to the aspect ratio of the electron beam pattern, resulting in a lower SNR for this preliminary experiment. Furthermore, Figure S6 provides experimental confirmation of the scaling of the signal with 1/|**R**|^2^ (and of *N*_min_ with |**R**|^2^).

In the parallel-beam LAD configuration, the beam diameter in the sample plane is approximately 30 μm, giving an upper bound on the spatial resolution of our SPINEM experiments. Figure 3(a) presents a scanning electron microscope (SEM) image, marking the probe positions (1–3) examined using the SPINEM technique. By evaluating the beam deflection γ_max_(**R**, ω) for a given probe position **R**, we sense the impact of the dynamic field **B**_⊥,dyn_(**r**, ω, *t*) at ω*t* = 0 on the electrons. Fields at this MW phase are defined as **B**_⊥,max_(**r**, ω) and **B**_∥,max_(**r**, ω). Position (1) corresponds to the same measurement as shown in Figure 2. For each position (1–3), the pattern length changes slightly due to small changes in the magnetic field strength of the microresonator coil delivered to each location. Therefore, we adjust the MW power at each location to optimize the SPINEM sensitivity.

**Figure 3:**
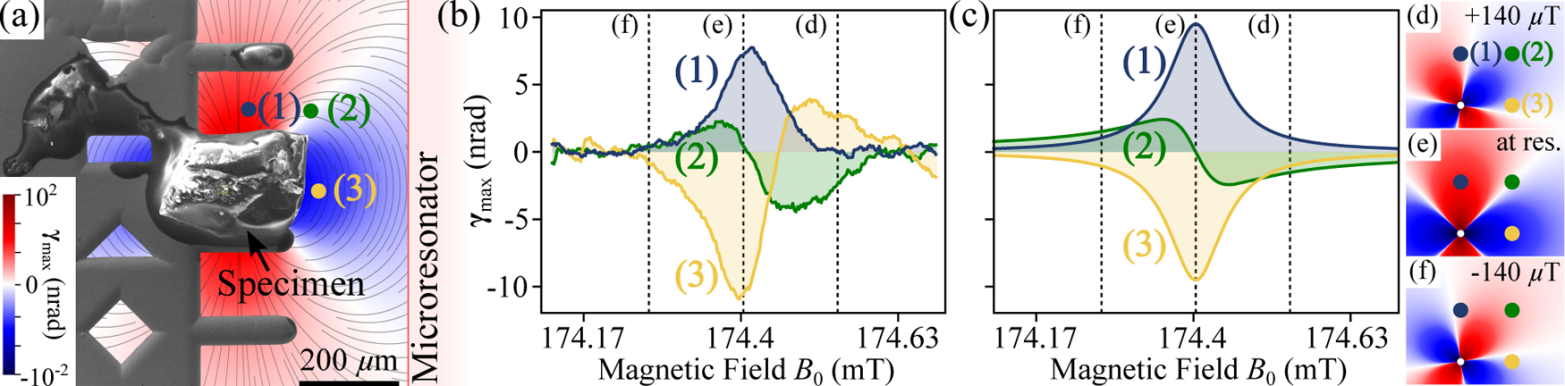
Spatially resolved SPINEM. (a) SEM image of the specimen mounted on a FIB lift-out grid. The microresonator position is highlighted in light red. The image is overlaid with exemplary magnetic dipole field lines of the specimen and with calculated beam deflection γ_max_ at resonance and for MW phase ω_res_ *t* = 0. (b) SPINEM spectra for different probe positions (1–3). The detected tilt angle is converted into beam deflection γ_max_, directly linked to the spin-electron interaction. Positions (1) and (3) exhibit lineshapes similar to c-ESR absorption spectra, whereas position (2) is reminiscent of ESR dispersion spectra. Since all measurements are referenced to the same **B**_1_ field of the driving MWs, the beam deflection γ_max_ serves as a measure of the in-plane dynamic field **B**_⊥,max_(**r**, ω) generated by the magnetization of the specimen, as the *B*_0_ biasing field varies. (c) Calculations for γ_max_, for positions (1–3), chosen to reflect the experimental arrangement in (a). Overall, the experiment and calculations are in good agreement. Panels (d–f) show 2D maps of calculated γ_max_ at +140, 0, and −140 μT *B*_0_ detuning from resonance at ω_res_ *t* = 0. MW generator output power was set to *P*_g_ = 24 dBm (1), *P*_g_ = 22 dBm (2) and *P*_g_ = 21 dBm (3). Other SPINEM parameters as in Figure 2.

The extracted spectra for the other positions qualitatively reproduce typical ESR spectra with dispersion (2) and absorption (3) features, see Figure 3(b). Deflection γ_max_(**R**, ω), which is proportional to **B**_⊥,max_(**r**, ω), is maximized at the resonance (|**B**_0_| = 174.4 mT) for positions (1) and (3), whereas it is zero for (2). While the dynamic magnetic field of the specimen is strongest at resonance, in the case of (2), |**B**_⊥,max_^(2)^| = 0 and |**B**_∥,max_^(2)^| is maximized, and thus γ_max_^(2)^ = 0. Furthermore, note that **γ**_max_^(3)^ = −**γ**_max_^(1)^, therefore **B**_⊥,max_^(3)^ = −**B**_⊥,max_^(1)^. As expected, far off the resonance, |ω – ω_res_| ≫ 0, the in-plane dynamic field |**B**_dyn_| decays to zero for all positions, therefore γ_max_^(1–3)^ → 0. For visualization, Figure 3(a) is overlaid with the calculated deflection γ_max_ for a MW phase of ω_res_ *t* = 0, ±2π,...

Figure 3(c) shows calculations of γ_max_ across the resonance at electron probe positions (1–3), chosen to reflect the probe positions as shown in Figure 3(a). Although detailed information on environmental effects and the sample’s composition, size, and past radiation-induced damage is missing, the calculations show excellent agreement with the experimental data. Analogously to the calculated 2D map presented in Figure 3(a), further calculated maps are plotted at *B*_0_ detunings of +140, 0, and −140 μT from resonance, see Figure 3(d–f). These maps reflect variations in both magnetization amplitude *m* and phase θ. The latter indicates the degree to which the spin system magnetization **m** lags behind the driving MW field **B**_1_. Figures S6 and S7 further demonstrate the mapping of the |**B**_⊥,max_|-induced deflections at different electron probe positions **R** around the specimen, representing a linescan away from the sample and a linescan along the length of the sample. Both provide a good agreement with theoretical predictions.

Overall, our results demonstrate the ability to both precisely control the spin state of a specimen (as demonstrated by the consecutive field-frequency map shown in Figure S8) and detect its response to a MW-drive using a free-space electron probe in a TEM. SPINEM enables the investigation of spin systems with excitation energies on the order of *E* ≈ 20 μeV and allows us to retrieve specimen properties, such as the gyromagnetic ratio, as demonstrated in Figure S8. Such sensitivity to low-energy magnetic excitations is beyond the reach of conventional TEM techniques such as EELS.

In the current proof-of-principle implementation, we are able to observe a clear signal even with a spin polarization, ∝ ω/*T*, of only ∼4 × 10^–4^ at 4.89 GHz and 290 K. Further adjustments to the setup can also increase the obtainable signal by improving the spin polarization. Lowering the sample temperature to liquid nitrogen or liquid helium temperatures^78,79^ can dramatically increase the signal, as well as decrease electron beam damage from the electron probe.^80–82^ The implementation of SPINEM using a high-frequency resonator would also enable the TEM OL to work at higher *B*_0_ magnetic field strengths, where lenses are optimized for high-resolution imaging and the spin polarization is stronger. Incorporating advanced STEM techniques such as DPC imaging or 4D-STEM could further enhance the SPINEM signal and push the spatial resolution toward the atomic scale. If a similar deflection sensitivity can be maintained for a 100 pm electron probe, even single spin contributions could become accessible in electron microscopy studies.^36,37^

## Conclusion

In this study, we have successfully developed the SPINEM technique, establishing the theoretical framework and technical foundations required for future advancements. Our implementation integrates a customized ESR setup with a modified TEM sample holder and specially optimized TEM settings, enabling the MW excitation of spin systems and their subsequent detection with a free-space electron probe. At resonance, we observe a beam deflection of γ_max_ = 7.5 nrad due to spin-electron interactions, with an unprecedented detection sensitivity reaching down to 290 prad. The recorded signal maps the projected dynamic magnetic vector field with a spatial resolution of approximately 30 μm, comparable to other spin mapping techniques such as BLS,^14–17^ while offering clear potential for further enhancement.

SPINEM thus extends both the capabilities of the TEM and the scope of spin detection beyond that of conventional ESR. With continued development, the integration of SPINEM spectroscopy with TEM imaging, diffraction, and analytical spectroscopy could enable truly correlative, spin-sensitive, and structural characterization at the nanoscale, all within a single instrument.

The results presented herein highlight the potential of SPINEM for various research fields, including possible extensions to other spin-probing techniques such as FMR or NMR. One potential application is the mapping of magnons and collective spin excitations in 2D layered thin films and nanostructures,^83^ enabling atomic-scale investigations relevant to spintronics and magnonics.^61^ Our technique also holds promise in the analysis of electron-induced radiation damage^80,84^ and the probing of sensitive samples^81,85–88^ without direct electron-specimen interaction. Finally, the position-encoded temporal information could enable time-resolved studies far beyond the capabilities of conventional electron detectors, without the use of sophisticated ultrafast TEM equipment. This work paves the way for the next generation of CW MW-pump electron-probe methodologies for the study of quantum materials, spin-based information processing, and magnetization dynamics on the nanoscale.

## Methods

### Experimental Section

Data are collected using a standard FEI Tecnai F20 TEM operated with continuous illumination and a beam current of 3.9 pA. We utilize LAD mode at a nominal camera length of 600 m. This highly parallel beam configuration yields a beam diameter of approximately 30 μm in the specimen plane. To accurately resolve the beam profile in LAD mode, we utilize a 4K CMOS Gatan Rio camera with a high pixel count of 4096 × 4096 and a minimum acquisition time of 50 ms (≈2.4 × 10^8^ precession cycles at the selected MW frequency). As a specimen, we use a spin-active radical: α,γ-bisdiphenylene-β-phenylallyl (BDPA)^67^ of ∼110 × 150 × 240 μm^3^ size. BDPA, also known as the Koelsch radical, is widely used in ESR as a benchmark sample^89^ due to its availability, stability at room temperature, narrow line width, and high spin density, *N*_s_ ≈ 1.5 spin/nm^3^.

For the SPINEM measurement, a CW MW-pump and electron-probe scheme is employed, with the electron beam precisely positioned aloof to the edge of the BDPA specimen. The setup employs a Rohde and Schwarz SMB100B MW generator to drive transitions between the specimen’s spin states. The CW frequency is set to ν = 4.89 GHz, while the magnetic field is finely tuned by varying the OL excitation in steps of 0.0001% (2.2 μT). However, sweeping the OL excitation disrupts the beam (e.g., parallelism). To compensate for this, the excitation of the condenser lens is dynamically adjusted to maintain spatial and momentum resolution. This is implemented via custom scripting of the TEM. For the c-ESR spectrum measurement, we use a setup similar to,^6^ with a Stanford Research SR810 lock-in amplifier.

### Image Processing

Each data point presented in the spectra in the figures is averaged over four individual images, each with a 5-s exposure time. To mitigate beam drifts, a diffraction shift correction was applied in the TEM’s projection system in between subsequent images, utilizing a center-of-mass (COM) analysis. In total, each spectrum in Figure 3 represents 976 individual images, amounting to a total data size of 64 GB.

To process the individual images, we first apply a 3 × 3 median filter and a global threshold to suppress camera noise. Next, a COM method is used to refine the center position with subpixel precision. Assuming that the deflections **α**, **β**, and **γ** at MW phases of 0, ±2π,..., have the same magnitude but opposite directions at ±π,..., this COM acts as a robust reference point. Relative to this center, we perform a principal component analysis (PCA) to evaluate the pattern tilt *ε* and hence the deflection γ. For more details, see SI B.1.

Using the tilt angle *ε* estimated via the PCA technique, we can determine changes in the electron beam pattern length. The image is projected onto an axis aligned with the angle ε, producing a deflection profile characterized by two prominent peaks, see Figure S1­(a). By fitting the position of these peaks with subpixel precision using a Gaussian model, we extract the change in the pattern length, see Figure S1­(b). The total pattern length is given by 2·(α_max_ + β_max_), see SI B.2.

### Signal Fitting

The observed offset in our analysis results from sweeping the OL excitation to tune the spin transitions across the resonance. This adjustment induces a slight rotation and magnification of the LAD pattern, which in our setup can only be corrected during postprocessing. To account for this, a polynomial (offset) combined with a Gaussian (absorption signal) and Gaussian derivative (dispersion signal) fit is performed to extract the deflections of the electron beam caused by spin precession. We use a second-degree polynomial for the *ε* and a fourth-degree polynomial for the β fit. Although the signal is expected to follow a Lorentzian line shape, the Gaussian fit provides a better match, suggesting a faster signal decay in the spectral tails, possibly introduced by the measurement technique.

### Calculations

For the calculations of the beam deflections, **α**, **β** and **γ**, induced by the dynamic magnetic fields **B**_1_ and **B**_dyn_, we follow the SI A and B. We consider a point-like spin system and a point-like electron probe positioned 150 μm away. We determine that the spin system contains *N* = 2.7 × 10^15^ spins, which produces the best fit with the experimentally measured values of beam deflections. Note that the specimen in the experiment, with an effective radius of 95 μm and a spin density of *N*_s_ = 1.5 spin/nm^3^, contains an estimated total of *N* = 5.9 × 10^15^ spins. For discussions on the discrepancy, see SI B.

The bias magnetic field is *B*_0_ = 0.17 T and the specimen’s temperature *T* = 290 K. At these conditions, only ∼10^–4^ of the spins are thermally polarized. To model the specimen’s dynamic in-plane magnetization and the magnetic field response, we use literature values for the BDPA specimen, *T*_1_ = (270 ± 40) ns and *T*_2_ = (120 ± 30) ns,^12,13^ and an experimentally estimated MW driving field |**B**_1,max_| = (20 ± 4) μT. This value matches the field produced by the microresonator at the impedance match (frequency ν = 4.89 GHz) for a MW generator output power of *P*_g_ = 24 dBm. At 200 keV, the electron velocity is v_e_ = 0.7 *c*, where *c* is the speed of light in vacuum.

To compute the images (Figure 1(b)), we calculate the deflection of the electron probe for |**B**_1,max_| = 20 μT over the MW driving phase interval ω*t* ∈ [0, 2π], followed by the application of a 2D Gaussian blur with a standard deviation of σ = 0.42 μrad. This produces a deflection pattern with |**α**_max_| = 16.75 μrad.

## Supplementary Material



## Notes

Beam deflection profiles (Figure S1), microresonator impedance match measurement (Figure S2), magnetic and electric field simulations (Figure S3), MW power vs SPINEM sensitivity (Figure S4), SPINEM with an LM-STEM probe (Figure S5), SPINEM signal distance scaling (Figure S6), SPINEM position line-sweep along the specimen (Figure S7), SPINEM field-frequency map (Figure S8) and Sections (beam deflection **α** (SI A); beam deflections **β**, **γ** (SI B); E-field contributions to the beam deflection (SI C); spin sensitivity estimates (SI D)) (PDF)
