# Supplementary material for: Sensing Spin Precession with Free Electrons
Source: ACS Nano. 2026 Jan 20;20(4):3435–43. doi: 10.1021/acsnano.5c13351 (PMC12875030; doi:10.1021/acsnano.5c13351)
Supplement: Supplementary file 1 [file nn5c13351_si_001.pdf]

Supporting Information: Sensing Spin Precession with Free Electrons

Antonín Jaroš, Michael S. Seifner, Johann Toyfl, Benjamin Czasch, Santiago Beltrán Romero, Isobel C. Bicket, and Philipp Haslinger*

Vienna Center for Quantum Science and Technology, Atominstitut, USTEM, Technische Universität Wien, Stadionallee 2, Vienna, 1020, Austria

E-mail: philipp.haslinger@tuwien.ac.at

Figure S1: Beam Deflection Profiles

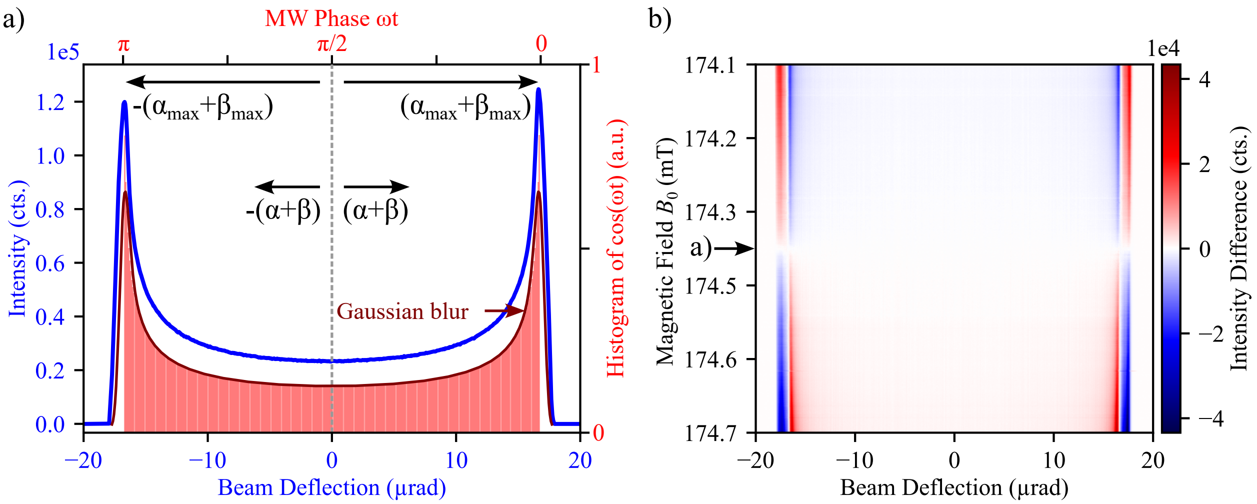

Figure S1: **Beam Deflection Profiles**. (a) The image of the electron beam pattern at resonance (B_0 = 174.4 mT) is projected onto an axis oriented at the angle ε, producing an intensity profile along the long axis of the pattern (blue curve). This profile displays two pronounced peaks characteristic of a cosine-like beam deflection. The total pattern length $(2 \cdot (\alpha_{\max} + \beta_{\max}))$ is obtained for each image by sub-pixel fitting of the peak positions. For comparison, the histogram of a $\cos(\omega t)$ function is shown in red, and its Gaussian-blurred form in dark red, illustrating strong similarities to the measured beam profile. (b) Difference profiles at various magnetic fields are shown relative to the resonant case in (a). At lower fields, the pattern, and thus the profile, is stretched, reflecting a redistribution from the center to the periphery. This stretching behavior is dominated by changes to the image magnification, caused by the OL excitation sweep, representing the background signal. The background is subtracted to isolate the specimen-induced contribution, attributed to the β deflection. Dataset is identical to Fig. 2.

Figure S2: Microresonator Impedance Match Measurement

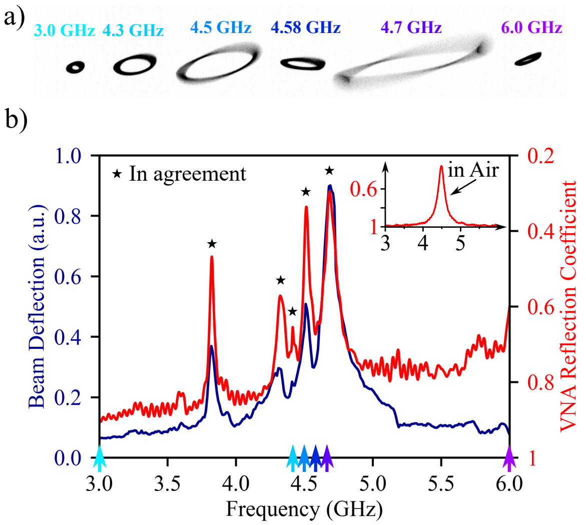

Figure S2: **Microresonator Impedance Match Measurement.** (a) Electron beam profiles at different driving frequencies, as indicated by the arrows in (b). (b) Different MW driving frequencies deliver varying power to the microresonator, resulting in changes of the $\mathbf{B}_{1,\max}$ field and beam deflection $\boldsymbol{\alpha}_{\max}$ as a function of frequency (shown in blue). Maximum beam deflection occurs at the microresonator's impedance match. For comparison, power delivery was also measured conventionally using a vector network analyzer (VNA) (in red). Both measurements show good agreement, although intensity variations are observed. The inset shows the VNA measurement of the microresonator conducted in air. Additional peaks observed in the TEM environment are attributed to parasitic capacitances and additional resonance modes induced by the TEM environment, acting as a metallic cavity.

Figure S3: Magnetic and Electric Field Simulations

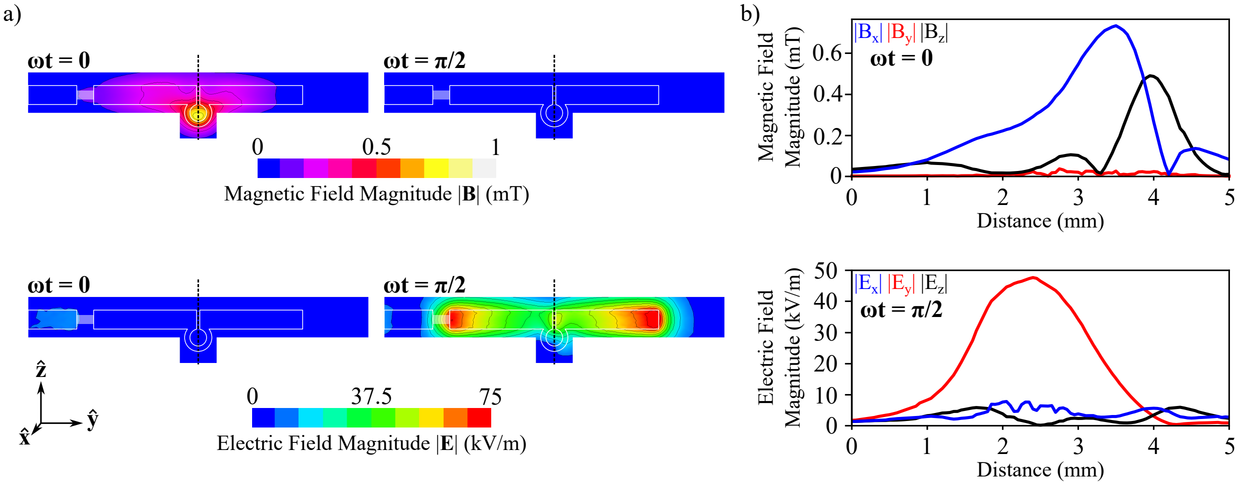

Figure S3: **Magnetic and Electric Field Simulations.** (a) Magnetic (top row) and electric (bottom row) field magnitude maps at MW phases $\omega t = 0$ (left column) and $\omega t = \pi/2$ (right column), evaluated 400 µm above the PCB surface. The maps illustrate the oscillatory behavior of both fields with the MW phase. The magnetic field $|\mathbf{B}|$ is strongly localized around the microcoil, whereas the undesired electric field $|\mathbf{E}|$ extends towards the upper region of the microresonator. (b) Corresponding magnetic (top) and electric (bottom) field profiles, showing the maximum fields present across all phases, along the dashed black line in (a), representing the electron trajectory in the TEM. Both the magnetic B_x and electric E_y field components contribute to deflecting the electron beam in the same direction, $(0, \alpha, 0)$, but with phase-shifted dependence on the MW drive. Integrating these field components along the electron flight path yields $\frac{\int E_\mathrm{y} dz}{\int v_\mathrm{e} B_\mathrm{x} dz} \approx 33\%$, indicating that the electric field contribution to electron beam deflection is not entirely negligible but is both phase-shifted relative to the magnetic field and significantly weaker in magnitude. Finally, since the maximum deflection α_max generated by the MW driving field serves only as a reference for determining the specimen-induced deflections γ_max and β_max, the residual influence of the electric field does not adversely affect the measurement evaluations presented in the main text. See SI C for more details. The field distributions are computed using Ansys HFSS (High Frequency Structure Simulator) for an input power of 4 W under optimal microresonator impedance matching conditions.

Figure S4: MW Power vs SPINEM Sensitivity

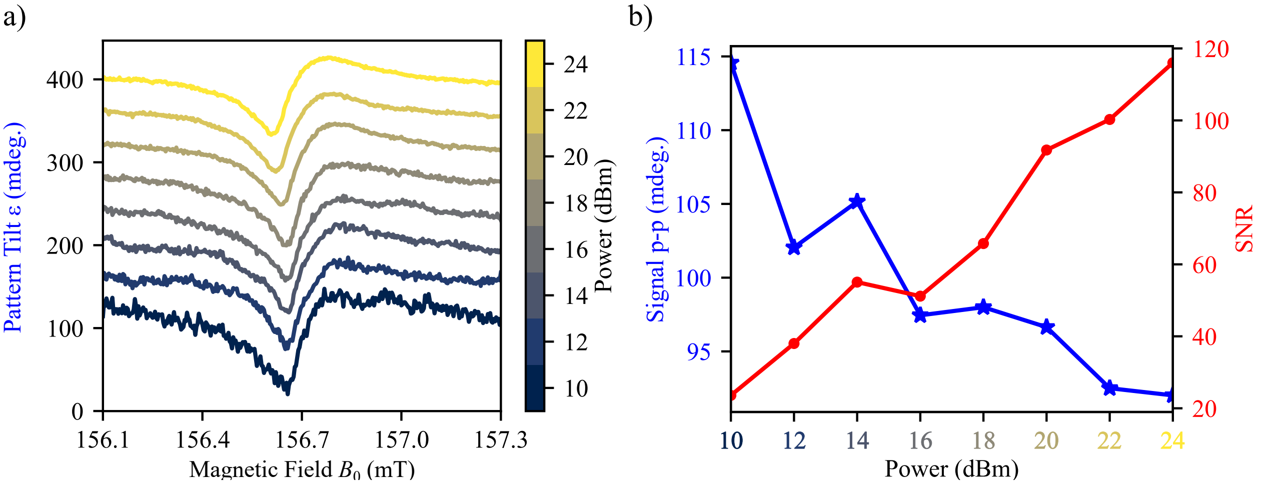

Figure S4: **MW Power vs SPINEM Sensitivity**. (a) Background-subtracted pattern tilts $\varepsilon(\omega)$ across the resonance, recorded under identical experimental conditions, but with varying MW generator output powers. Each spectrum is offset by 40 mdeg. along the vertical axis. (b) The extracted peak-to-peak (p-p) signal amplitude (blue) vs the SNR (red). As the MW power increases, the measured signal diminishes, indicating spin system driving with excessive power, *i.e.*, $\mathbf{B}_1(t)$ field. However, the overall sensitivity of the SPINEM measurement improves, as the PCA-based evaluation of $\varepsilon(\omega)$ becomes more reliable with increasingly elongated electron beam patterns induced by stronger $\mathbf{B}_1(t)$. For different positions around the specimen, the pattern length can change slightly, requiring an adjustment of MW power to optimize sensitivity at the selected camera length. Note that the BDPA specimen used in this measurement differs in shape and size from that presented in the main text body. The higher SNR observed here, compared to Fig. 2, arises from the larger specimen volume, providing a correspondingly stronger magnetization and spin-induced deflection signal. Experimental conditions: MW frequency 4.38 GHz, camera acquisition time 0.5 s per spectral point, camera binning 4 × 4, beam current 37.5 pA, nominal camera lengths: 410 m (10-18 dBm), 275 m (20 dBm), 220 m (22-24 dBm).

Figure S5: SPINEM With an LM-STEM Probe

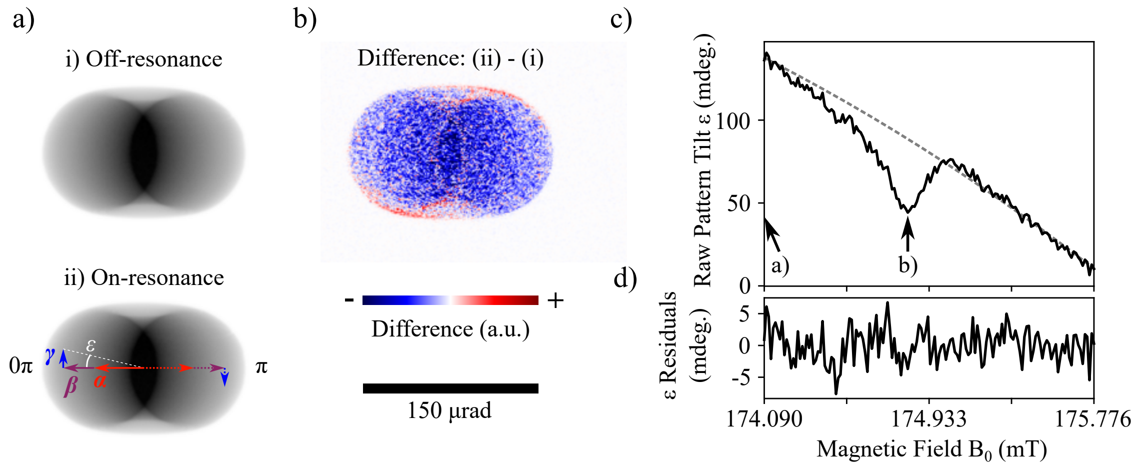

Figure S5: **SPINEM With an LM-STEM Probe** Using the condenser lens, the electron beam was focused at the sample plane, mimicking a low-magnification (LM)-STEM probe. (a) represents the images of the angular distribution of the electron beam at 8 m nominal camera length, off (i) and on resonance (ii), respectively. $\boldsymbol{\alpha}, \boldsymbol{\beta}, \boldsymbol{\gamma}$ vectors mark the deflection from the $\mathbf{B}_1$ driving field, and the dynamic magnetic field generated by the specimen at the resonance. Compared to the image of the parallel beam (Fig. 2(c)), taken at a nominal camera length of 600 m, a considerable overlap between the deflection at a MW phase of 0π and π can be observed. While this complicates the evaluation of the pattern length change, one can still reliably extract the ε tilting. The difference image (b) highlights ε tilting at resonance. (c) Recorded SPINEM spectrum for such conditions, demonstrating the possibility of high spatial resolution measurements using a focused electron probe. The (a)(i,ii) images are corrected for the background polynomial tilt (dashed gray line in (c)) and centered using a COM method. (d) reveals the ε fit residuals, with a standard deviation of 2.6 mdeg. Note that the BDPA specimen used in this measurement differs in shape and size from that presented in the main text body. Moreover, the MW setup and the microresonator also differ from those employed in the main experiment. Experimental conditions: MW frequency 4.905 GHz, MW output power $P_g = 21$ dBm, camera acquisition time 2 s per spectral point, camera binning 4×4, beam current 113.6 pA.

Figure S6: SPINEM Signal Distance Scaling

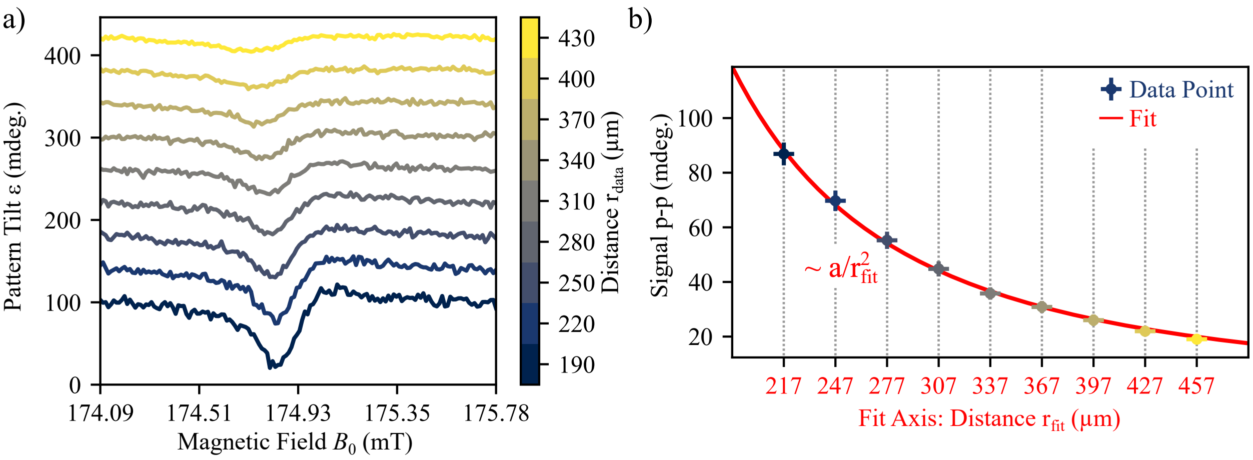

Figure S6: **SPINEM Signal Distance Scaling.** (a) Background-subtracted pattern tilts $\varepsilon(\omega)$ across the resonance, recorded under identical experimental conditions, but with varying electron beam probe position r_{data} from the volumetric center of the specimen. Specimen dimensions and the volumetric centre were estimated from images of different sample projections acquired with SEM. Each spectrum is offset by 40 mdeg. along the vertical axis. (b) The extracted p-p signal amplitudes (data points) at each position, fitted with the expected $\sim a/r_{\text{fit}}^2$ scaling (red curve), are consistent with the expected scaling of a point-like dipole field. a is an amplitude scaling constant. Note that there is an offset between r_{data} and r_{fit} of 27 µm, which can be attributed to the differences between the real specimen features and the approximation of the specimen as a point-like magnetic dipole. However, overall, the data are in good agreement with the theoretically predicted scaling. Note that the BDPA specimen used in this measurement differs in shape and size from that presented in the main text body. Experimental conditions: MW frequency 4.905 GHz, MW output power $P_{\text{g}} = 21$ dBm, camera acquisition time 2 s per spectral point, camera binning 4×4, beam current 113.6 pA, nominal camera length 8 m.

Figure S7: SPINEM Position Line-sweep Along the Specimen

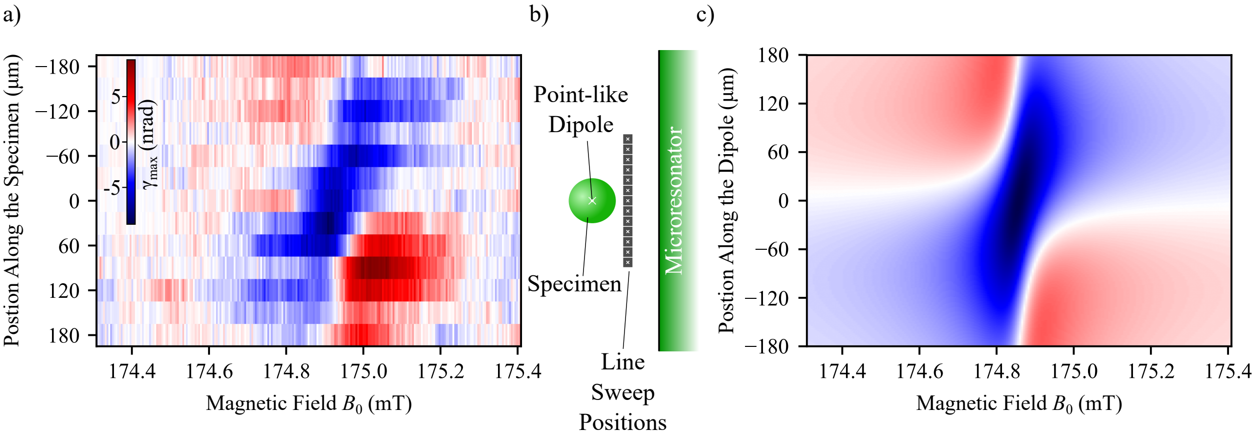

Figure S7: **SPINEM Position Line-Sweep Along the Specimen.** (a) Background-subtracted electron beam deflection γ_{max} across the resonance, recorded under identical experimental conditions, but with varying electron beam probe position around the specimen, as illustrated in the sketch in (b). This measurement is analogous to conventional 4D-STEM, in which the electron probe is scanned across the specimen and a diffraction pattern is recorded at each probe position. The SPINEM experiment adds a fifth dimension: a sweep across the specimen's resonance. These measurements demonstrate our ability to map the dynamic magnetic fields generated by the specimen at different probe positions, highlighting the potential of this technique for nanoscale mapping of individual particles and probing their resonances individually. (c) Calculations of electron beam deflections (in a.u.) due to the dynamic magnetic fields of a point-like magnetic dipole, representing our specimen. The measurement and calculations are in excellent agreement, although deviations can be observed, mainly attributed to the measurement noise and the lack of knowledge about the morphology of our specimen, spin distribution, and past beam-induced damage. Note that the BDPA specimen used in this measurement differs in shape and size from that presented in the main text body. Experimental conditions: MW frequency 4.905 GHz, MW output power $P_{\text{g}} = 18$ dBm, camera acquisition time 2 s per spectral point, camera binning 4×4, beam current 480 pA, parallel beam with a nominal camera length of 40 m.

Figure S8: SPINEM Field-Frequency Map

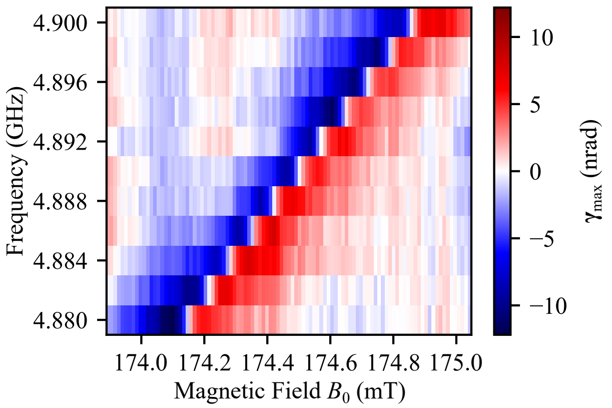

Figure S8: **SPINEM Field-Frequency Map.** Background-subtracted electron beam deflection γ_{max}, acquired by sweeping the magnetic field for each microwave frequency. This measurement illustrates our ability to precisely control and detect the state of the spin system. As the biasing B_0 field generated by the OL polepiece is varied, the signal shifts according to the gyromagnetic ratio of the specimen, following the relation: $\nu = \Gamma B_0$, where $\Gamma = \frac{1}{2\pi}\frac{g_e \mu_B}{\hbar}$. Here, g_e is the electron's g-factor, μ_B is the Bohr magneton, and $\hbar$ is the reduced Planck constant. We evaluate the gyromagnetic ratio of our specimen as $\Gamma = (27.9 \pm 1.2)$ GHz/T, which matches well with the literature values of BDPA.[S1–S3] Our SPINEM measurements allow for the investigation of spin systems with an excitation energy of $E \approx 20$ µeV. Experimental settings: MW output power $P_g = 21$ dBm. Other parameters as in Fig. 2.

Supporting Information

SI A: Beam Deflection α

The introduction of the dynamic MW field $\mathbf{B}_1(\omega, t)$ results in a deflection of the electron beam. The beam profile appears as a line or an ellipse (Fig. S2(a)), depending on the linear or elliptical polarization of the $\mathbf{B}_1$ field created by the microresonator. At the microresonator impedance match, the $\mathbf{B}_1$ field at the centre of a circular microcoil along its $\hat{\mathbf{x}}$-axis is given by:

$$\mathbf{B}_1 = \hat{\mathbf{x}} \frac{\mu_0 a^2}{2(a^2 + x^2)^{\frac{3}{2}}} I \cos(\omega t), \tag{1}$$

with a the coil radius and x the distance along the coil axis and $I \cos(\omega t)$ the AC current. At the location of our specimen d, and in the small area δd, where our measurements are conducted, the variance of the field can be estimated as:

$$\frac{\Delta B}{B} \approx \left| \frac{1}{B} \frac{\mathrm{d}B}{\mathrm{d}x} \right|_{x=d} \delta d = \frac{3d}{a^2 + d^2} \delta d. \tag{2}$$

Hence, provided

$$\frac{3d}{a^2 + d^2} \delta d \ll 1, \tag{3}$$

the field can be considered highly homogeneous within the measurement region δd:

$$\mathbf{B}_1 = \hat{\mathbf{x}} \frac{\mu_0 a^2}{2(a^2 + d^2)^{\frac{3}{2}}} I \cos(\omega t) = \mathbf{B}_{1,\max} \cos(\omega t) \tag{4}$$

The electron travels along a perpendicular path, defined by the $\hat{\mathbf{z}}$-axis. The field is confined by the region defined by the microcoil radius a:

$$\mathbf{B}_1(z, \omega, t) = \begin{cases} \mathbf{B}_{1,\max}(\omega) \cos(\omega t), & z \in [-a, a] \\ 0, & \text{otherwise} \end{cases} \tag{5}$$

Now, to model the deflection of the electron beam by the oscillating $\mathbf{B}_1(z, \omega, t)$ magnetic field, we evaluate the Lorentz force acting on the electrons along their flight path:

$$\mathbf{F}(z, \omega, t) = -e\mathbf{v}_\mathrm{e} \times \mathbf{B}_1(z, \omega, t) \Rightarrow m_\mathrm{e}^* \frac{d\mathbf{v}}{dt} = \mathbf{F}, \tag{6}$$

where $\mathbf{v}_\mathrm{e} = (0, 0, -v_\mathrm{e}) = (0, 0, -0.7c)$ is the velocity vector of the electron beam, e is the electron charge, and m_e^* is a relativistic mass. The transverse velocity components of the initial electron probe are neglected due to the high longitudinal velocity of the 200 keV electrons. Furthermore, we neglect the contributions from the electric fields. For more details on this, see SI C. Integrating in time, we get the speed of the electrons $\mathbf{v}(\omega, t)$ after the interaction:

$$\mathbf{v}(\omega, t) = \mathbf{v}_e + \frac{1}{m_\mathrm{e}} \int_{t_1}^{t_2} \mathbf{F}(z(t'), \omega, t') dt' \tag{7}$$

The change of the velocity in longitudinal direction can be considered negligible, $v_\mathrm{z}(t') \approx -v_\mathrm{e}$, therefore:

$$dt' = \frac{dz}{v_z(t')} \approx -\frac{dz}{v_e} \Rightarrow t' \approx t - \frac{L}{v_e} \tag{8}$$

$$\mathbf{v}(\omega, t) \approx \mathbf{v}_e - \frac{1}{m_\mathrm{e} v_\mathrm{e}} \int_{-\infty}^{\infty} \mathbf{F}(z, \omega, t - \frac{z}{v_e}) dz \tag{9}$$

The magnetic field, i.e., the Lorentz force, changes harmonically with time, but the fast nature of the electron allows us to use the quasi-static approximation. The corresponding timescales of the electron-specimen and electron-microcoil interaction are:

$$\frac{L_\mathrm{specimen}}{v_e} \sim 1 \text{ ps} < \frac{L_\mathrm{microcoil}}{v_e} \sim 5 \text{ ps} \ll \frac{2\pi}{\omega} \sim 200 \text{ ps}, \tag{10}$$

where $L_\mathrm{specimen} \approx 200$ µm and $L_\mathrm{microcoil} \approx 1$ mm are the effective interaction lengths of the electron with the specimen and microcoil, respectively. The phase of the MWs changes by $\sim 0.05\pi$ before the electron fully passes the microcoil, and by $\sim 0.01\pi$ before passing the specimen. Hence, the quasi-static approximation can be considered a highly reasonable

assumption. Therefore:

$$\mathbf{F}(z,\omega,t') = \mathbf{F}(z,\omega,t-\frac{z}{v_e}) \approx \mathbf{F}(z,\omega,t) \tag{11}$$

Now we can compute the deflection of the electron beam, $\boldsymbol{\delta}_{\rm dyn}(\omega,t)$. For small transverse velocity change, $\Delta\mathbf{v}_{\perp} = \mathbf{v} - \mathbf{v}_{\rm e}$, we get:

$$\boldsymbol{\delta}_{\rm dyn}(\omega,t) = \arctan\frac{\Delta\mathbf{v}(\omega,t)}{v_e} \approx \frac{1}{m_e v_e^2}\int_{-\infty}^{\infty}\mathbf{F}(z,\omega,t)dz \tag{12}$$

where we used a small-angle approximation. The equation is in a general form that can be used for both the deflection of the beam caused by the microcoil and the dynamically driven specimen. Substituting from Eq. 5 and using the Lorentz equation 6, we get:

$$\boldsymbol{\delta}_{\rm dyn}(\omega,t) = (0,\alpha,0) = -\frac{e}{m_{\rm e}^{\star}v_{\rm e}^2}\int_{-\infty}^{+\infty}\mathbf{v}_{\rm e}\times\mathbf{B}_{1,\rm max}(z,\omega)\cos(\omega t)\,dz. \tag{13}$$

The α amplitude follows:

$$\alpha(\omega,t) = \frac{el}{m_{\rm e}^{\star}v_{\rm e}}B_{1,\rm max}(\omega)\cos(\omega t). \tag{14}$$

Here, l is the spatial extent of the B_1 field. For the interaction with both the microcoil and the specimen fields, the deflection would follow $\boldsymbol{\delta}_{\rm dyn}(\omega,t) = (\gamma,\alpha+\beta,0)$. For the derivation of β and γ deflections, see SI B.

Here, the deflection α reflects the power delivered by the MWs from the microresonator. By analyzing the beam pattern spread, $\alpha_{\rm max}$, when no specimen is present, we can evaluate the power delivery and assess the impedance match of the microresonator,[S4] as shown in Fig. S2(b) (blue curve).

For comparison, impedance matching was also measured using a vector network analyzer (VNA), as shown in Fig. S2(b) (red curve). A VNA evaluates the power delivered to the

device under test (DUT) against the power reflected from it, with a reflection coefficient of 0 representing a perfect impedance match, where all power is transferred to the DUT.

The impedance match of our microresonator alone is shown in the inset of Fig. S2(b). When the microresonator is inserted into the holder and placed inside the TEM, additional peaks appear. This is attributed to parasitic capacitances introduced by the proximity of additional metal components to the microresonator's conductors. Moreover, the TEM itself acts as a metallic cavity, introducing further resonance conditions. The higher background observed in the VNA measurements inside the TEM is likely due to calibration inconsistencies, as the VNA calibration procedure is performed in air before insertion of the holder into the TEM. This movement of components after the calibration procedure can degrade calibration accuracy.

Overall, both the TEM beam deflection analysis and the VNA measurements show good agreement. Intensity variations arise as the VNA captures the power delivered to the entire DUT, whereas the beam measurement is only locally sensitive to the $\mathbf{B}_1$ field, theoretically enabling highly spatially resolved mapping of power delivery to specific regions of the DUT.[S4]

SI B: Beam Deflections β, γ

For $\mathbf{B_1}(\omega \approx \omega_{\text{res}})$, the specimen's magnetization vector $\mathbf{M}(\omega, t)$ begins to precess, resulting in static out-of-plane component $\mathbf{s}(\omega)$ and dynamic in-plane component $\mathbf{m}(\omega, t)$, see Fig. 1. The electron beam deflection can be modeled by determining the in-plane magnetization of the specimen, achieved by solving the Bloch equation. The in-plane magnetization vectors can be described as:

$$m^{\|\mathrm{B}_1}(\omega, t) = M_{x'}(\omega)\cos(\omega t) - M_{y'}(\omega)\sin(\omega t), \tag{15}$$

$$m^{\perp\mathrm{B}_1}(\omega, t) = M_{y'}(\omega)\cos(\omega t) + M_{x'}(\omega)\sin(\omega t), \tag{16}$$

Here, $m^{\|\mathrm{B}_1}$ and $m^{\perp\mathrm{B}_1}$ denote the components of the in-plane magnetization vectors that are, respectively, collinear and perpendicular to the linear polarization of the driving magnetic field $\mathbf{B}_1(\omega, t) = (B_1, 0)$. $M_{x'}$ and $M_{y'}$ are the magnetization components in a rotating frame of reference around the $\mathbf{B}_0$-axis at the frequency of the driving MWs, expressed as:

$$M_{x'}(\omega) = \frac{(\omega - \omega_{\text{res}})(2\pi\Gamma)(\frac{B_1}{2})T_2^2}{1 + [(\omega - \omega_{\text{res}})T_2]^2 + (2\pi\Gamma)^2(\frac{B_1}{2})^2 T_1 T_2} M_0, \tag{17}$$

$$M_{y'}(\omega) = \frac{(2\pi\Gamma)(\frac{B_1}{2})T_2}{1 + [(\omega - \omega_{\text{res}})T_2]^2 + (2\pi\Gamma)^2(\frac{B_1}{2})^2 T_1 T_2} M_0, \tag{18}$$

where $\Gamma = \frac{1}{2\pi}\frac{g_{\mathrm{e}}\mu_{\mathrm{B}}}{\hbar}$ is the gyromagnetic ratio of the sample, and T_1 and T_2 are the corresponding relaxation times in the parallel and transverse directions relative to the static spin alignment. In conventional ESR, the components $M_{x'}(\omega)$ and $M_{y'}(\omega)$ are measured to determine the parameters ω_{res}, Γ, T_1, and T_2, which provide valuable information about the specimen. The measurements of $M_{x'}(\omega)$ and $M_{y'}(\omega)$ for varying frequency or bias magnetic field B_0 across the resonance represent the ESR dispersion and absorption spectra, respectively.

In comparison to the main text, it is apparent that $m(\omega) = \sqrt{M_{x'}^2(\omega) + M_{y'}^2(\omega)}$ and

$\theta(\omega) = -\arctan(\frac{M_{x'}(\omega)}{M_{y'}(\omega)}) - 90°$. Therefore, $m^{\parallel \mathrm{B}_1}(\omega, t) = m\cos(\omega t + \theta)$ and $m^{\perp \mathrm{B}_1}(\omega, t) = m\sin(\omega t + \theta)$.

We consider a spin-active specimen modeled as a point source, with a point-like electron probe positioned nearby. The electron beam is deflected by the dynamic magnetic near-field $\mathbf{B}_{\mathrm{dyn}}(\mathbf{r}, \omega, t)$, generated by the dynamic magnetization $\mathbf{M}(\omega, t)$ of the specimen:

$$\mathbf{B}_{\mathrm{dyn}}(\mathbf{r}, \omega, t) = \frac{\mu_0}{4\pi r^3}\left[\frac{3(\boldsymbol{\mu}\cdot\mathbf{r})\mathbf{r}}{r^2} - \boldsymbol{\mu}\right], \tag{19}$$

where $\boldsymbol{\mu}(\omega, t) = \mathbf{M}(\omega, t)V$ is the dipole moment of the sample, V is the expected sample volume, and $\mathbf{r} = (x, y, z)$ represents the electron probe position coordinates relative to the specimen. The xy-plane indicates the sample plane, while the z-axis aligns with the TEM optical axis and with the $\mathbf{B}_0$ field.

As electrons interact with the sample, the Lorentz force causes an angular deflection of the electron beam:

$$\mathbf{F}(\mathbf{r}, \omega, t) = -e\mathbf{v}_{\mathrm{e}} \times \mathbf{B}_{\mathrm{dyn}}(\mathbf{r}, \omega, t). \tag{20}$$

Here, $\mathbf{B}_{\mathrm{dyn}}(\mathbf{r}, \omega, t) = (B_{\parallel,\mathrm{dyn}}, B_{\perp,\mathrm{dyn}}, B_{\mathrm{z,dyn}})$, where $B_{\parallel,\mathrm{dyn}}$ and $B_{\perp,\mathrm{dyn}}$ are the dynamic field components parallel and perpendicular to the alignment of $\mathbf{B}_1(\omega, t) = (B_1, 0, 0)$.

Our SPINEM measurements (Fig. 2-4) provide a measure of angular deflection of the electron beam, obtained by integrating the Lorentz force (Eq. 20) acting on the electrons over their flight path:[S5]

$$\boldsymbol{\delta}_{\mathrm{dyn}}(\mathbf{R}, \omega, t) \approx \frac{-e}{m_{\mathrm{e}}^{\star} v_{\mathrm{e}}^2}\int_{-\infty}^{+\infty} \mathbf{v}_{\mathrm{e}} \times \mathbf{B}_{\mathrm{dyn}}(\mathbf{r}, \omega, t)\, dz. \tag{21}$$

$\boldsymbol{\delta}_{\mathrm{dyn}}(\mathbf{R}, \omega, t) \approx (\gamma, \beta, 0)$ can be interpreted as an average measure of the dynamic magnetic fields, $B_{\parallel,\mathrm{dyn}}$ and $B_{\perp,\mathrm{dyn}}$. The resulting beam deflection due to specimen-electron interaction follows:

$$\beta(\mathbf{R}, \omega, t) = \mathcal{N}\frac{y^2 m^{\parallel \mathrm{B}_1} - 2xym^{\perp \mathrm{B}_1} - x^2 m^{\parallel \mathrm{B}_1}}{(x^2 + y^2)^2}, \tag{22}$$

$$\gamma(\mathbf{R},\omega,t) = \mathcal{N}\frac{y^2 m^{\perp \mathrm{B}_1} + 2xym^{\parallel \mathrm{B}_1} - x^2 m^{\perp \mathrm{B}_1}}{(x^2+y^2)^2} \tag{23}$$

where $\beta(\mathbf{R},\omega,t)$ and $\gamma(\mathbf{R},\omega,t)$ represent deflection parallel and orthogonal to the deflection $\alpha(\omega,t)$. $\mathcal{N} = \frac{-e}{m_{\mathrm{e}}^{\star} v_{\mathrm{e}}}\frac{\mu_0 V}{2\pi}$ represents a pre-factor composed of various constants (μ_0 is vacuum magnetic permeability). Taking Eq. 23 as an example, it can be rearranged as a linear combination of absorption, $M_{y'}(\omega)$, and dispersion, $M_{x'}(\omega)$, spectra:

$$\begin{aligned}\gamma(\mathbf{R},\omega,t) = &\mathcal{N}\Big[\mathcal{A}(\mathbf{R})M_{y'}(\omega) + \mathcal{B}(\mathbf{R})M_{x'}(\omega)\Big]\cos(\omega t) \\ &+\mathcal{N}\Big[\mathcal{A}(\mathbf{R})M_{x'}(\omega) - \mathcal{B}(\mathbf{R})M_{y'}(\omega)\Big]\sin(\omega t),\end{aligned} \tag{24}$$

where $\mathcal{A}(\mathbf{R}) = \frac{y^2-x^2}{(x^2+y^2)^2}$ and $\mathcal{B}(\mathbf{R}) = \frac{2xy}{(x^2+y^2)^2}$. As a consequence, γ can therefore be interpreted as a measure of conventional absorption and dispersion spectra. Rearranging Eq. 24 into its simplest form, it can be shown that γ represents a sinusoidal deflection of the electron beam:

$$\gamma(\mathbf{R},\omega,t) = \mathcal{N}m(\omega)\mathcal{C}(\mathbf{R})\sin(\omega t + \theta(\omega) + \phi(\mathbf{R})), \tag{25}$$

where $\mathcal{C}(\mathbf{R}) = \frac{1}{R^2}$ and $\phi(\mathbf{R}) = \arctan(\frac{2xy}{y^2-x^2})$. $\phi(\mathbf{R})$ can be interpreted as an additional phase lag. Similarly to Eq. 24, we can rewrite Eq. 22 into:

$$\begin{aligned}\beta(\mathbf{R},\omega,t) = &\mathcal{N}\Big[\mathcal{A}(\mathbf{R})M_{x'}(\omega) - \mathcal{B}(\mathbf{R})M_{y'}(\omega)\Big]\cos(\omega t) \\ &-\mathcal{N}\Big[\mathcal{A}(\mathbf{R})M_{y'}(\omega) + \mathcal{B}(\mathbf{R})M_{x'}(\omega)\Big]\sin(\omega t).\end{aligned} \tag{26}$$

Using the aforementioned equations 26 and 24 and assuming a specific phase ($\omega t = n\pi$, $n = 0, 1, 2, ...$) where the deflections are maximum, $\beta_{\max}(\mathbf{R},\omega)$ and $\gamma_{\max}(\mathbf{R},\omega)$, it can be shown how different positions $\mathbf{R}$ of the beam result in varying $\beta_{\max}$ and $\gamma_{\max}$ deflections. This results in a measurement of absorption and/or dispersion ESR spectrum. For the special case where $x = 0$ and $y \neq 0$, which reflects the electron beam position (1) in Fig. 3, the

calculated deflection is given by:

$$\gamma_{\max}(\mathbf{R},\omega) = \frac{\mathcal{N}}{y^2} M_{y'}(\omega), \tag{27}$$

$$\beta_{\max}(\mathbf{R},\omega) = \frac{\mathcal{N}}{y^2} M_{x'}(\omega). \tag{28}$$

Similarly, for $x \neq 0$ and $y = 0$, reflecting position (3) in Fig. 3,

$$\gamma_{\max}(\mathbf{R},\omega) = -\frac{\mathcal{N}}{x^2} M_{y'}(\omega), \tag{29}$$

$$\beta_{\max}(\mathbf{R},\omega) = -\frac{\mathcal{N}}{x^2} M_{x'}(\omega). \tag{30}$$

A change in beam position from (1) to (3) is equivalent to a MW phase shift of π at the first mixing stage, as utilized in conventional ESR. For $x = y \neq 0$, reflecting beam position (2) in Fig. 3, a measurement of $\gamma_{\max}$ results in a measurement of an ESR dispersion spectrum (and likewise an absorption spectrum from a measurement of β):

$$\gamma_{\max}(\mathbf{R},\omega) = \frac{\mathcal{N}}{2x^2} M_{x'}(\omega), \tag{31}$$

$$\beta_{\max}(\mathbf{R},\omega) = -\frac{\mathcal{N}}{2x^2} M_{y'}(\omega). \tag{32}$$

These equations demonstrate a complex, yet predictable interplay of beam deflections $\beta_{\max}$ and $\gamma_{\max}$ and the electron probe position $\mathbf{R}$. At other positions, the measured deflections represent a linear superposition of absorption and dispersion spectra, see Eq. 22, 23.

To resolve γ and β experimentally, we would require a high sensitivity for the electron beam deflection as well as a time resolution better than $1/4.89\ \text{GHz}^{-1} \approx 204$ ps at the MW frequency $\nu = 4.89$ GHz. Our TEM is operated with a continuous electron beam and a conventional CMOS detector, with which it is not possible to directly resolve either $\beta_{\max}$ and $\gamma_{\max}$, or β and γ at a specific time t. Each electron beam pattern, acquired over 5 seconds, is a sum of many precession periods ($5\ \text{s} \cdot 4.89\,\text{GHz} = 24.45 \cdot 10^9$).

SI B.1: Experimental Evaluation of $\gamma_{\mathbf{max}}$

To resolve γ experimentally, we focus on the change in the electron pattern tilt ε as the B_0 field is swept across the resonance:

$$\tan\varepsilon(\mathbf{R},\omega,t) = \frac{\gamma(\mathbf{R},\omega,t)}{\alpha(\omega,t)+\beta(\mathbf{R},\omega,t)} \approx \frac{\gamma(\mathbf{R},\omega,t)}{\alpha(\omega,t)}. \tag{33}$$

To the acquired images, we apply the PCA algorithm for angle estimation. PCA operates by averaging over the coordinates of each image and subsequently estimating a direction of maximum variance in the intensity distribution:

$$\tan\varepsilon(\mathbf{R},\omega) = \frac{v_y^{(\max)}}{v_x^{(\max)}}, \tag{34}$$

where $(v_x^{(\max)}, v_y^{(\max)})$ are the x and y components of the principal eigenvector $\boldsymbol{v}^{(\max)}$. For each image, corresponding to a specific MW frequency ω, this can be reflected in Eq. 33 by the time-average of the electron beam deflection over a single precession cycle from 0 to 2π, with phase ωt:

$$\tan\varepsilon(\mathbf{R},\omega) = \frac{1}{2\pi}\int_0^{2\pi} \frac{\gamma(\mathbf{R},\omega,t)}{\alpha(\omega,t)}\, d(\omega t). \tag{35}$$

Using $\alpha(\omega,t) = \alpha_{\max}\cos(\omega t)$ and substituting Eq. 24 into Eq. 35, we obtain:

$$\begin{aligned}\tan\varepsilon(\mathbf{R},\omega) = \frac{\mathcal{N}}{2\pi}\int_0^{2\pi} \Big\{\Big[\frac{\mathcal{A}(\mathbf{R})}{\alpha_{\max}} M_{y'}(\omega) + \frac{\mathcal{B}(\mathbf{R})}{\alpha_{\max}} M_{x'}(\omega)\Big] \\ + \Big[\frac{\mathcal{A}(\mathbf{R})}{\alpha_{\max}} M_{x'}(\omega) - \frac{\mathcal{B}(\mathbf{R})}{\alpha_{\max}} M_{y'}(\omega)\Big] \tan(\omega t)\Big\}\, d(\omega t).\end{aligned} \tag{36}$$

Since $\int_0^{2\pi}\tan(\omega t)\, d(\omega t) = 0$, we get:

$$\tan\varepsilon(\mathbf{R},\omega) = \mathcal{N}\Big[\frac{\mathcal{A}(\mathbf{R})}{\alpha_{\max}} M_{y'}(\omega) + \frac{\mathcal{B}(\mathbf{R})}{\alpha_{\max}} M_{x'}(\omega)\Big] = \frac{\gamma_{\max}}{\alpha_{\max}}, \tag{37}$$

where $\gamma_{\max}(\mathbf{R},\omega) = \mathcal{N}\Big[\mathcal{A}(\mathbf{R})M_{y'}(\omega) + \mathcal{B}(\mathbf{R})M_{x'}(\omega)\Big]$. In this way, the time dependence of ε is eliminated and we obtain a static measure of specimen properties at a MW phase ($\omega t = 0, \pm 2\pi, \dots$). In Fig. 3-4, we use Eq. 37 to compute the electron beam deflection $\gamma_{\max}$ caused by the interaction with the spin system: $\gamma_{\max} = \alpha_{\max} \tan\varepsilon$.

This measurement is influenced only by the position of the electron probe $\mathbf{R}$ and the B_0 field, which determines whether the resonance condition is satisfied. To demonstrate the position dependency, calculated 2D deflection maps of $\gamma_{\max}$ are shown in Fig. 3(d-f), at +140 mT, 0 mT, and -140 mT detuning from resonance, respectively.

To estimate the expected value of $\gamma_{\max}$ for our measurements, we use conditions reflecting those in the conducted experiments. A point-like electron probe is positioned at $y = 0$ and $x = 150$ µm from a point-like spin system. The spin system contains an estimated $N = 5.9 \cdot 10^{15}$ spins, equivalent to that in a spherical specimen with a radius $r_{\mathrm{s}} = 95$ µm and a spin density of $N_{\mathrm{s}} = 1.5$ spin/nm^3. This volume effectively matches the sample used in the experiment, as measured by projections acquired in an SEM. Static magnetization follows $M_0 = N_{\mathrm{s}} \frac{\mu_{\mathrm{B}}^2 B_0}{k_{\mathrm{B}} T}$ (μ_0 is vacuum magnetic permeability, μ_{B} is the Bohr magneton and K_{B} is the Boltzmann constant), where magnetic field $B_0 = 0.17$ T, and temperature $T = 290$ K. At these conditions, only $\sim N_{\mathrm{s}} \cdot 10^{-4}$ of the spins are thermally polarized. To model the specimen's dynamic in-plane magnetization and the magnetic field response, we use literature values for the BDPA specimen, $T_1 = 270$ ns and $T_2 = 120$ ns,[S6] and an experimentally estimated MW driving field $|\mathbf{B}_{1,\max}| = 20$ µT.

For 200 keV electrons, traveling at $v_{\mathrm{e}} = 0.7c$, these conditions result in $\gamma_{\max} = 20$ nrad at resonance. This value is larger than the experimentally measured one ($\gamma_{\max,\,\mathrm{exp}} \approx 7.5$ nrad). Using instead $N = 2.7 \cdot 10^{15}$ spins for the calculations results in the best fit with the experimentally measured values. This discrepancy is likely due to the assumptions made about the point-like nature of the electron probe or the estimation of the specimen's magnetic dipole field. Additionally, we lack precise knowledge about the specimen's morphology, spin distribution, and the potential beam-induced damage after long TEM experiments at 200

keV and currents of 3.9 pA.

SI B.2: Experimental Evaluation of β_{max}

In order to resolve β, we projected the recorded images onto an axis aligned with the angle ε. This produces a deflection profile characterized by two prominent peaks, see Fig. S1. We directly fit to the pattern peaks with sub-pixel precision and extract the total pattern length. Variations to this pattern length correspond to the time-independent $\alpha_{\text{max}} + \beta_{\text{max}}$ deflections:

$$
\begin{aligned}
2 \cdot [\beta_{\text{max}}(\mathbf{R}, \omega) + \alpha_{\text{max}}(\omega)] &= [\beta_{\text{max}}(\mathbf{R}, \omega) + \alpha_{\text{max}}(\omega)] \Big|_{\omega t = 0} \\
&\quad - [\beta_{\text{max}}(\mathbf{R}, \omega) + \alpha_{\text{max}}(\omega)] \Big|_{\omega t = \pi} \\
&= 2 \cdot \left\{ \mathcal{N} \left[\mathcal{A}(\mathbf{R}) M_{x'}(\omega) - \mathcal{B}(\mathbf{R}) M_{y'}(\omega) \right] + \frac{el}{m_{\text{e}}^{\star} v_{\text{e}}} B_{1,\text{max}} \right\}, \qquad (38)
\end{aligned}
$$

where $\beta_{\text{max}}(\mathbf{R}, \omega) = \mathcal{N} [\mathcal{A}(\mathbf{R}) M_{x'}(\omega) - \mathcal{B}(\mathbf{R}) M_{y'}(\omega)]$. For $|\omega - \omega_{\text{res}}| \gg 0$, $\beta_{\text{max}}(\mathbf{R}, \omega) \to 0$, allowing us to isolate and subtract the contribution of $\alpha_{\text{max}}(\omega)$. By performing a fit to the SPINEM spectra recorded across the resonance, we can therefore separate the total pattern length into a contribution from the spin system, $\beta_{\text{max}}(\mathbf{R}, \omega)$, and one from the MW driving field, $\alpha_{\text{max}}(\omega)$.

SI C: E-field Contributions to the Beam Deflection

In addition to magnetic interactions, the electron beam may also experience deflection due to electric fields generated by either the microresonator or the specimen. In this section, we analyze these additional electric-field-induced effects and derive the corresponding contribution to the electron beam deflection. The resulting expressions will be compared with the magnetic deflection $\boldsymbol{\delta}_{\text{dyn}}$ previously obtained in SI A and SI B.

SI C.1: Microresonator E-fields

For the first approximation, we restrict our analysis to the electromagnetic field within the microresonator region, where spatial variations of the driving field are negligible, allowing it to be approximated as a locally homogeneous time-varying magnetic field:

$$\mathbf{B}_1 \approx \hat{\mathbf{x}} B_{1,\text{max}} \cos(\omega t). \tag{39}$$

For such a field, we can compute the near-axis magnetic potential as:

$$\mathbf{A}_1 \approx \frac{1}{2}\mathbf{B}_1 \times \mathbf{r}_\perp = \frac{1}{2}(0, -zB_{1,\text{max}}\cos(\omega t), yB_{1,\text{max}}\cos(\omega t)), \tag{40}$$

where $\mathbf{r}_\perp = (0, y, z)$. Hence:

$$\mathbf{E}_1 \approx -\frac{\partial \mathbf{A}_1}{\partial t} - \nabla\varphi_\text{E} = \frac{1}{2}(0, -z\omega B_{1,\text{max}}\sin(\omega t), y\omega B_{1,\text{max}}\sin(\omega t)). \tag{41}$$

The divergence of $\mathbf{E}_1$ for $y, z \to \pm\infty$ arises from the near-axis approximation used for $\mathbf{A}_1$, which neglects the full spatial structure of the electromagnetic field outside the microresonator region. This assumption further prevents us from integrating over the entire flight path of the electron, $z \in (-\infty, +\infty)$, but at the same time reveals that the $\mathbf{E}_1 \cdot \hat{\mathbf{x}}$ is zero and $\mathbf{E}_1 \cdot \hat{\mathbf{y}}$ is odd around $z = 0$, i.e., the integration across this region is always zero as well. The effect of $\mathbf{E}_1 \cdot \hat{\mathbf{z}} = y\omega B_{1,\text{max}} \sin(\omega t)$ is equivalent to a defocusing of the electron beam.

Furthermore, while the $\mathbf{B}_1$ magnetic contribution to the deflection of the beam is maximal at a MW phase of $\omega t = 0, \pm\pi, \pm 2\pi, \ldots$ (at the extrema of the deflection pattern), the $\mathbf{E}_1$ electric contribution at this phase is always zero. The strongest effect of the electric fields is at a MW phase of $\omega t = \pm\frac{\pi}{2}, \pm\frac{3\pi}{2} \ldots$, i.e., at the center of the deflection pattern.

To properly assess and compare the relative influence of magnetic and electric fields on electron beam deflection, we performed electromagnetic simulations of our microresonator using Ansys HFSS (High-Frequency Structure Simulator). The simulated field distributions, shown in Fig. S3(a), reveal the spatial distribution and phase-dependent oscillation of the MW magnetic and electric fields 400 µm away from the surface of the PCB. The magnetic field $|\mathbf{B}|$ is strongly confined to the vicinity of the microcoil, while the electric field $|\mathbf{E}|$ extends toward the upper section of the microresonator. The electric field vector is dominated by its y-component.

The corresponding field profiles along the dashed black line in Fig. S3(b), representing the electron trajectory in the TEM, allow for a direct comparison between the magnetic (B_x) and electric (E_y) components. Both fields act to deflect the electron beam in the same direction, $\boldsymbol{\delta}_\text{dyn} = (0, \alpha, 0)$, but with a phase offset. Integrating these components along the electron flight path yields:

$$\frac{\hat{\mathbf{y}} \cdot \Delta\mathbf{v}_\text{E-field}(\omega t = \pi/2)}{\hat{\mathbf{y}} \cdot \Delta\mathbf{v}_\text{B-field}(\omega t = 0)} = \frac{\int E_\text{y} dz}{\int v_\text{e} B_\text{x} dz} \approx 33\%. \tag{42}$$

This ratio represents the worst-case scenario and reveals that the electric-field contribution, although not entirely negligible, is weaker and out of phase with the magnetic-field-induced deflection, which is consistent with our previous estimations. Moreover, while B_x is strongly localized within the microcoil region, coinciding with the specimen position and the TEM eucentric plane where deflection sensitivity is maximal, the peak of E_y occurs approximately 1 mm above the microcoil, well out of the focal plane of the electron lenses. Consequently, the electron beam is significantly less sensitive to this offset E_y field.

In such a worst-case scenario, the main effect of the electric field would be a small systematic error in the extracted deflections, $\gamma_{\max}$ and $\beta_{\max}$. Specifically, the electric field contribution introduces a phase offset between the electron beam deflection and the precession phase of the specimen's magnetization, leading to a partial mixing of absorption and dispersion components for a given probe position $\mathbf{R}$. A similar effect may also arise from the quasi-static approximation, as the MW field phase slightly evolves during the finite electron transit time across the specimen region. A more detailed analysis of these minor effects is out of the scope of this proof-of-principle demonstration. The experimental data remain in excellent agreement with the theoretical predictions, with any small deviations plausibly attributable to electric-field contributions. Future refinements of the method could further mitigate these effects, either by reducing the electric field through redesign of the microresonator or by accounting for its influence more fully during SPINEM signal analysis. In contrast, a fully time-resolved MW pump–electron probe scheme could be employed in future implementations to reduce the time-averaged influence of both the microresonator's electric and magnetic fields on the electron beam patterns collected.

Finally, since the MW-induced deflection amplitude $\alpha_{\max}$ serves merely as a reference for extracting the specimen-induced deflections $\gamma_{\max}$ and $\beta_{\max}$, we conclude that the presence of the electric fields does not adversely affect our analysis in the main text.

SI C.2: Specimen E-fields

We now consider the effects of electric fields generated by the temporally fluctuating magnetic fields from the spin system. At the resonance of the specimen, we excite a time-varying magnetic dipole:

$$\mathbf{B}_{\mathrm{dyn}}(\mathbf{r}, \omega, t) = \nabla \times \mathbf{A}(\mathbf{r}, \omega, t) = \nabla \times \left[\frac{\mu_0}{4\pi} \frac{\boldsymbol{\mu}(\omega, t) \times \mathbf{r}}{r^3} \right]. \tag{43}$$

The electric field $\mathbf{E}_{\text{dyn}}(\mathbf{r}, \omega, t)$ can be estimated using the Maxwell equations:

$$\mathbf{E}_{\text{dyn}}(\mathbf{r}, \omega, t) = -\frac{\partial \mathbf{A}(\mathbf{r}, \omega, t)}{\partial t} - \nabla \varphi_{\text{E}} = -\frac{\mu_0}{4\pi} \frac{\dot{\boldsymbol{\mu}}(\omega, t) \times \mathbf{r}}{r^3}, \tag{44}$$

where $\dot{\boldsymbol{\mu}}(\omega, t) = \frac{\partial \boldsymbol{\mu}}{\partial t} \sim (\dot{m}^{\parallel \text{B}_1}, \dot{m}^{\perp \text{B}_1}, 0) = (\frac{\partial m^{\parallel \text{B}_1}}{\partial t}, \frac{\partial m^{\perp \text{B}_1}}{\partial t}, 0)$. We use the Coulomb gauge, setting $\nabla \varphi_{\text{E}} = 0$. The final velocity of the electron beam, after the interaction with the electric field, can be expressed as

$$\mathbf{v}(\mathbf{R}, \omega, t) \approx \mathbf{v}_{\text{e}} - \frac{e}{m_{\text{e}}^{\star} v_{\text{e}}} \int_{-\infty}^{+\infty} \mathbf{E}_{\text{dyn}}(\mathbf{r}, \omega, t)\, dz. \tag{45}$$

During the integration, the contributions from $\mathbf{E}_{\text{dyn}}(\mathbf{r}, \omega, t) \cdot \hat{\mathbf{x}}$ and $\mathbf{E}_{\text{dyn}}(\mathbf{r}, \omega, t) \cdot \hat{\mathbf{y}}$ vanish, resulting in changes only to the longitudinal speed. This change in velocity is given by

$$\Delta \mathbf{v} = \mathbf{v} - \mathbf{v}_{\text{e}} = (0, 0, \Delta v_{\text{z}}), \tag{46}$$

where

$$\Delta v_{\text{z}}(\mathbf{R}, \omega, t) = \mathcal{N} \frac{\dot{m}^{\parallel \text{B}_1} y - \dot{m}^{\perp \text{B}_1} x}{x^2 + y^2} \tag{47}$$

with the same prefactor $\mathcal{N}$ as in Eq. (23). Hence, the dynamic electric field $\mathbf{E}_{\text{dyn}}(\mathbf{r}, \omega, t)$ changes the electron beam energy by an amount too small to be detected in electron energy loss spectroscopy, effectively introducing a phase-locked focus blur in the final image through the chromatic aberrations of the objective lens. This effect, however, is minor and was not observed in our measurements.

We consider a specific MW phase $\omega t = 0, \pm\pi, \pm 2\pi, \ldots$ corresponding to the maximum change in the velocity, $\Delta v_{\max}$:

$$\Delta v_{\max}(\mathbf{R}, \omega) = \mathcal{N} \omega \frac{M_{x'} x + M_{y'} y}{x^2 + y^2}. \tag{48}$$

The appearance of ω arises from the time derivative of the specimen's magnetization in

Eq. (44). The ratio of the longitudinal speed change to the initial electron speed is:

$$\kappa_{\max}(\mathbf{R},\omega) = \frac{\Delta v_{\max}(\mathbf{R},\omega)}{v_{\mathrm{e}}} = \frac{\mathcal{N}\omega}{v_{\mathrm{e}}} \frac{M_{x'}x + M_{y'}y}{x^2+y^2}. \tag{49}$$

For the special case where $x = 0$ and $y \neq 0$, which reflects the electron beam position (1) in Fig. 3, the calculated $\kappa_{\max}$ ratio simplifies to

$$\kappa_{\max}(\mathbf{R},\omega) = \frac{\mathcal{N}\omega}{y v_{\mathrm{e}}} M_{y'}(\omega). \tag{50}$$

Similarly, for $x = y \neq 0$, reflecting position (2) in Fig. 3, we obtain

$$\kappa_{\max}(\mathbf{R},\omega) = \frac{\mathcal{N}\omega}{2x v_{\mathrm{e}}} [M_{x'}(\omega) + M_{y'}(\omega)]. \tag{51}$$

The dynamic electric field $\mathbf{E}_{\mathrm{dyn}}$ modifies only the longitudinal component of the electron velocity, whereas the dynamic magnetic field $\mathbf{B}_{\mathrm{dyn}}$ alters the transverse velocity, deflecting the electron beam by an angle $\boldsymbol{\delta}_{\mathrm{dyn}} = (\gamma, \beta, 0)$. It is therefore evident that the contribution of the electric field does not influence the experimental ε estimations presented in the main text, as ε depends solely on the transverse velocity change of the electron beam.

Moreover, $\kappa_{\max}(\mathbf{R},\omega)$ scales inversely with the specimen–probe distance, in contrast to the inverse quadratic dependence of $\gamma_{\max}(\mathbf{R},\omega)$ and $\beta_{\max}(\mathbf{R},\omega)$. For example, at $x = 0$, $y = 150$ µm (representing the probe position in Fig. 2), $\omega t = 0$, and $\frac{\omega}{v_{\mathrm{e}}} = \frac{2\pi \times 5 \times 10^9}{0.7c} \approx 150\ \mathrm{m}^{-1}$, the change in electron velocity induced by $\mathbf{E}_{\mathrm{dyn}}$ constitutes only a small fraction of that caused by $\mathbf{B}_{\mathrm{dyn}}$:

$$\frac{\hat{\mathbf{z}} \cdot \Delta \mathbf{v}_{\text{E-field}}}{\hat{\mathbf{x}} \cdot \Delta \mathbf{v}_{\text{B-field}}} \approx \frac{\kappa_{\max}}{\gamma_{\max}} = \frac{\frac{\mathcal{N}\omega}{y v_{\mathrm{e}}} M_{y'}(\omega)}{\frac{\mathcal{N}}{y^2} M_{y'}(\omega)} = \frac{\omega y}{v_{\mathrm{e}}} \approx 2\ \%. \tag{52}$$

Thus, under the present experimental conditions, the electric field from the sample's spin precession contributes only marginally to the overall electron beam deflection, compared to the dominant magnetic-field effect.

SI D: Spin Sensitivity Estimates

To benchmark the spin sensitivity N_{min} of a conventional ESR (c-ESR) spectrum[S7] against the SPINEM technique, we compare the underlying detection mechanisms. In a standard ESR setup, the precessing magnetization of the excited specimen induces a voltage in a microcoil, based on the principle of reciprocity.[S8] The induced voltage is subsequently read out via a lock-in detection scheme. In the SPINEM approach, the same precessing magnetization generates a deflection of the electron beam. While both methods rely on a magnetic response of the spins, the resulting signal amplitudes scale differently with system parameters. Understanding these scaling laws is therefore essential for evaluating the relative sensitivity and potential of the two techniques.

SI D.1: Conventional ESR

For the derivation of the spin sensitivity of a conventional MW setup, we follow Ref.[S9] and the principle of reciprocity.[S8] As derived in[S7], the detected ESR signal S for an arbitrary resonator follows:

$$S = \frac{\sqrt{2}}{2}\omega_0 m V \frac{2B_1}{\sqrt{P_{\mathrm{in}}}}\sqrt{R_0}, \tag{53}$$

where R_0 is the impedance of the MW system and P_{in} is the input power from the MW source. For an optimally driven specimen in a CW ESR experiment, the in-plane magnetization is $m = \frac{1}{2}\sqrt{\frac{T_2}{T_1}}M_0$. For simplicity, we assume $T_1 = T_2$; using exact values of these parameters will result in only minor variations of the final N_{min}, but will not affect the scaling laws derived herein.

Noise at the detector from antenna resistance with temperature T, limited to a bandwidth of Δf, is $N = \sqrt{4k_BT\left(\frac{R_0}{2}\right)\Delta f}$. Therefore, the SNR follows:

$$\mathrm{SNR} = \frac{S}{3N} = \frac{\omega_0 M_0 V \frac{B_1}{\sqrt{P_{\mathrm{in}}}}}{3\sqrt{4k_BT\Delta f}}, \tag{54}$$

and the theoretical spin sensitivity $N_{\min}$ of our antenna utilized in c-ESR measurement is:

$$N_{\min} = \frac{N_{\mathrm{s}} V}{\mathrm{SNR}\sqrt{\Delta f}} = \frac{24 (k_B T)^{3/2} \sqrt{R_0}}{B_0^2 (2\pi\Gamma)^3 \hbar^2 B_{\mathrm{u}}}, \tag{55}$$

where $B_{\mathrm{u}} = \frac{B_1}{I\cos(\omega t)}$ is a unitary magnetic field generated by the microcoil. The factor 3 in the SNR stems from a convention.

The unitary magnetic field at the centre of a circular microcoil along its axis is given by:

$$B_{\mathrm{u}} = \frac{\mu_0 a^2}{2(a^2 + x^2)^{\frac{3}{2}}}, \tag{56}$$

with a the coil radius and x the distance along the coil axis. Maximum sensitivity is obtained when the specimen is positioned at the coil center ($x \to 0$), where $B_{\mathrm{u}} = \frac{\mu_0}{2a}$. Table S1 summarizes the scaling dependencies:

Table S1: Scaling of c-ESR spin sensitivity $N_{\min}$ with key parameters. A lower $N_{\min}$ corresponds to higher sensitivity.

**Parameter**	**Scaling of $N_{\min}$**	**Origin**
Temperature T	$\propto T^{3/2}$	Thermal noise $\propto T^{1/2}$
		Spin polarization $\propto 1/T$
Bias field B_0	$\propto 1/B_0^2$	Zeeman splitting $\to$ spin polarization $\propto B_0$
		Faraday induction into the microcoil $\propto B_0$
Coil radius a	$\propto 1/a$	Smaller coils $\to$ larger B_{u}

SI D.2: SPINEM

The detection and analysis methods used in SPINEM differ significantly from those used in c-ESR. Importantly, we are analyzing two-dimensional images to extract changes in the rotation and length of the phase-locked electron beam pattern. In the analysis, we consider a set of N_{tot} independent observations $\mathbf{x}_i = (x_i, y_i)$ distributed in a two-dimensional plane. These data points cluster along two dominant directions, suggesting an underlying anisotropy that can be characterized using principal component analysis (PCA). The PCA procedure identifies the orthogonal directions (principal axes) that maximize and minimize the variance

of the data, respectively. The statistical covariance matrix is defined as

$$\mathbf{S} = \begin{pmatrix} S_{xx} & S_{xy} \\ S_{yx} & S_{yy} \end{pmatrix} = \frac{1}{N_{\text{tot}}} \sum_{i=1}^{N_{\text{tot}}} (\mathbf{x}_i - \bar{\mathbf{x}})(\mathbf{x}_i - \bar{\mathbf{x}})^T, \tag{57}$$

for data points $\mathbf{x}_i = (x_i, y_i)$ and statistical mean $\bar{\mathbf{x}} = (\bar{x}, \bar{y})$. The covariance is computed with respect to a fixed coordinate frame defined by the detector screen's arbitrary axes x and y.

The orientation of the first principal component, measured relative to the y-axis, is given by

$$\varepsilon = \frac{1}{2}\,\text{atan2}\big(2S_{xy},\, S_{yy} - S_{xx}\big). \tag{58}$$

Our goal is to determine the asymptotic variance $\text{Var}(\varepsilon)$ to leading order in $1/N_{\text{tot}}$, thereby quantifying the statistical uncertainty in the estimated orientation of the principal component. To do this, we apply a first-order linearization. The idea is to approximate ε by expanding it around the true, fixed population covariance matrix

$$\mathbf{\Sigma} = \begin{pmatrix} \Sigma_{xx} & \Sigma_{xy} \\ \Sigma_{xy} & \Sigma_{yy} \end{pmatrix}, \tag{59}$$

which represents the true covariance structure of the random vector $\mathbf{x}_i$. While $\mathbf{\Sigma}$ is a fixed (non-random) parameter describing the population, the statistical covariance $\mathbf{S}$ fluctuates around it due to finite-sampling noise. Thus, we can linearize $\varepsilon(\mathbf{S})$ around $\mathbf{\Sigma}$ using a first-order Taylor expansion:

$$\varepsilon - \varepsilon_{\text{m}} \approx \underbrace{\left.\frac{\partial \varepsilon}{\partial S_{xx}}\right|_{\mathbf{S}=\mathbf{\Sigma}}}_{d_{xx}} (S_{xx} - \Sigma_{xx}) + \underbrace{\left.\frac{\partial \varepsilon}{\partial S_{yy}}\right|_{\mathbf{S}=\mathbf{\Sigma}}}_{d_{yy}} (S_{yy} - \Sigma_{yy}) + \underbrace{\left.\frac{\partial \varepsilon}{\partial S_{xy}}\right|_{\mathbf{S}=\mathbf{\Sigma}}}_{d_{xy}} (S_{xy} - \Sigma_{xy}). \tag{60}$$

Here, ε_{m} is the true angle estimate ($N_{\text{tot}} \to \infty$), d_{xx}, d_{yy}, and d_{xy} denote the partial derivatives of ε with respect to the statistical covariance elements, evaluated at the true covariance

matrix $\mathbf{\Sigma}$. This expression provides an approximate linear relationship between the random fluctuations of the statistical covariances and the estimation error in the angle ε. Using Eq. (58), the derivatives take the explicit form

$$d_{xx} = \frac{v}{2a}, \quad d_{yy} = -\frac{v}{2a}, \quad d_{xy} = \frac{u}{a}, \tag{61a}$$

where we define $u := \Sigma_{yy} - \Sigma_{xx}$, $v := 2\Sigma_{xy}$ and $a := u^2 + v^2$.

This first-order Taylor expansion, Eq. 60, approximates the nonlinear function $\varepsilon(\mathbf{S})$ by a linear combination of the sample covariance deviations. Using the standard property that the variance of a linear combination is determined by the coefficients and covariances of the inputs, we obtain:

$$\mathrm{Var}(\varepsilon) \approx \sum_{p,q\in\{xx,yy,xy\}} d_p d_q \,\mathrm{Cov}(S_p, S_q). \tag{62}$$

For Gaussian data, the joint distribution of the statistical covariance matrix is well understood and leads to a closed-form expression for the covariances between its elements. Specifically, if the data vectors are independent realizations from a bivariate normal distribution $\mathbf{x}_i \sim \mathrm{Norm}(\mathbf{x}_\mathrm{m}, \mathbf{\Sigma})$, then the scaled sample covariance matrix follows a *Wishart distribution*, denoted by

$$N_\mathrm{tot}\mathbf{S} \sim W_2(\mathbf{\Sigma}, N_\mathrm{tot} - 1),$$

for dimensionality 2.[S10] From the second-order moments of the Wishart distribution (see[S10]), one obtains:

$$\mathrm{Cov}(S_{ij}, S_{kl}) = \frac{1}{N_\mathrm{tot}}\big(\Sigma_{ik}\Sigma_{jl} + \Sigma_{il}\Sigma_{jk}\big), \qquad i,j,k,l \in \{x,y\}. \tag{63}$$

This expression describes how the elements of the statistical covariance matrix co-vary across repeated random samples.

Employing the expressions for the covariances of the statistical covariance elements in Eq. (63) together with the derivative coefficients in Eq. (61), we obtain the following asymp-

totic approximation for the variance of the tilting angle:

$$\mathrm{Var}(\varepsilon) \approx \frac{\Sigma_{xx}\Sigma_{yy} - \Sigma_{xy}^2}{N_{\mathrm{tot}}\left[(\Sigma_{yy} - \Sigma_{xx})^2 + 4\Sigma_{xy}^2\right]}. \tag{64}$$

The $1/N_{\mathrm{tot}}$ factor indicates the usual sampling improvement with larger data sets. An alternative and often more intuitive representation uses the eigenvalues of the covariance matrix $\mathbf{\Sigma}$. Denoting the principal variances by $\lambda_+ \geq \lambda_-$, we have

$$\lambda_\pm = \frac{1}{2}\left(\Sigma_{xx} + \Sigma_{yy} \pm \sqrt{(\Sigma_{yy} - \Sigma_{xx})^2 + 4\Sigma_{xy}^2}\right). \tag{65}$$

From this, it follows that

$$\lambda_+\lambda_- = \det(\mathbf{\Sigma}) = \Sigma_{xx}\Sigma_{yy} - \Sigma_{xy}^2, \quad (\lambda_+ - \lambda_-)^2 = (\Sigma_{yy} - \Sigma_{xx})^2 + 4\Sigma_{xy}^2,$$

which respectively quantify the "volume" ($\lambda_+\lambda_-$) of the 2D point cloud and the separation ($(\lambda_+ - \lambda_-)$) between the principal component variances. Substituting these relations, the variance can equivalently be expressed as

$$\boxed{\mathrm{Var}(\varepsilon) \approx \frac{\lambda_+\lambda_-}{N_{\mathrm{tot}}\,(\lambda_+ - \lambda_-)^2}.} \tag{66}$$

This form provides a clear interpretation: the variance of the estimated tilting angle increases with the overall spread of the data, as measured by the product $\lambda_+\lambda_-$, and decreases as the separation between the principal component variances ($\lambda_+ - \lambda_-$) grows. In the limiting case of isotropic data, where $\lambda_+ \approx \lambda_-$, the variance diverges ($\mathrm{Var}(\varepsilon) \to \infty$), reflecting that the orientation of the principal axis becomes ill-defined.

Next, we model the deflection from the MW driving field; each observation is drawn from a Gaussian distribution whose mean depends on a phase parameter (ωt), representing, for

example, the phase-locked deflection. Specifically, we consider

$$\mathbf{x}_i \sim \mathrm{Norm}\big(\mathbf{x}_\mathrm{m}(\omega t),\, \Sigma\big), \qquad \mathbf{x}_\mathrm{m}(\omega t) = \begin{pmatrix} A\cos(\omega t) \\ B\cos(\omega t + \phi_d) \end{pmatrix},$$

where ϕ_d is a phase delay between the x and y components of $\mathbf{x}_\mathrm{m}$, while A and B represent the respective deflection amplitudes. We assume the phase ωt is sampled uniformly on $[0, 2\pi)$ (or equivalently, we average over ωt uniformly). Both the expectation value over ωt, $\mathbb{E}_{\omega t}[\cos(\omega t)] := \frac{1}{2\pi}\int_0^{2\pi} \cos(\omega t) d(\omega t) = 0$, and over the marginal mean are zero, $\mathbb{E}_{\omega t}[\mathbf{x}_\mathrm{m}(\omega t)] = 0$. In other words, the MW driving field does not shift the center-of-mass (COM) of the Gaussian distribution but instead introduces an additional contribution to its covariance. This property is crucial for the PCA analysis, as drifts or jumps of the COM (*e.g.*, due to environmental instabilities) do not affect the determination of the principal angle. Consequently, PCA inherently filters out such translational components.

By the law of total covariance[S11], the marginal covariance of the observation $\mathbf{x}_i$ is the sum of the within-cycle covariance and the covariance of the mean values taken over one full oscillation period of the driving phase ωt:

$$\mathbf{\Sigma}' \;=\; \mathbb{E}_{\omega t}[\mathrm{Cov}(\mathbf{x}_i \mid \omega t)] + \mathrm{Cov}_{\omega t}\big(\mathbf{x}_\mathrm{m}(\omega t)\big) \;=\; \mathbf{\Sigma} + \mathrm{Cov}_{\omega t}\big(\mathbf{x}_\mathrm{m}(\omega t)\big). \tag{67}$$

Since the covariance of the random variable $\mathbf{x}_i$ is independent of the phase parameter ωt, this covariance corresponds to $\mathbf{\Sigma}$ that arises from the measurement process. Meanwhile, the second term corresponds to:

$$\begin{aligned} \mathrm{Cov}_{\omega t}(\mathbf{x}_\mathrm{m}(\omega t)) =& \mathbb{E}_{\omega t}[\mathbf{x}_\mathrm{m}(\omega t)\mathbf{x}_\mathrm{m}(\omega t)^\top] = \mathbb{E}_{\omega t} \begin{pmatrix} A^2\cos^2\omega t & AB\cos^2\omega t \\ AB\cos^2\omega t & B^2\cos^2\omega t \end{pmatrix} \\ =& \frac{1}{2}\begin{pmatrix} A^2 & AB\cos(\phi_d) \\ AB\cos(\phi_d) & B^2 \end{pmatrix}. \end{aligned} \tag{68}$$

Hence, the effective population covariance is:

$$\mathbf{\Sigma}' = \mathbf{\Sigma} + \frac{1}{2}\begin{pmatrix} A^2 & AB\cos(\phi_d) \\ AB\cos(\phi_d) & B^2 \end{pmatrix} \tag{69}$$

which adds extra variance due to the data acquisition process.

If we use the eigenvalue expression for the variance of the tilt angle in Eq. (66), now evaluated for $\mathbf{\Sigma}'$, it is useful to define $\varsigma_1 = \operatorname{tr}\mathbf{\Sigma}'$ and $\varsigma_2 = \det\mathbf{\Sigma}'$. Then the eigenvalues $\lambda'_\pm$ (ordered $\lambda'_+ \geq \lambda'_-$) are the roots of $\lambda^2 - \varsigma_1\lambda + \varsigma_2 = 0$, hence

$$\lambda'_\pm = \frac{\varsigma_1}{2} \pm \frac{1}{2}\sqrt{\varsigma_1^2 - 4\varsigma_2}. \tag{70}$$

Explicitly,

$$\varsigma_1 = \operatorname{tr}\mathbf{\Sigma} + \tfrac{1}{2}(A^2 + B^2) = \Sigma_{xx} + \Sigma_{yy} + \tfrac{1}{2}(A^2 + B^2), \tag{71}$$

and, by direct expansion, the matrix determinant is

$$\begin{aligned} \lambda'_+\lambda'_- = \varsigma_2 \; &= \det\big(\mathbf{\Sigma} + \mathrm{Cov}_{\omega t}(\mathbf{x}_\mathrm{m}(\omega t))\big) \\ &= \det\big(\mathbf{\Sigma}\big) + \det\big(\,\mathrm{Cov}_{\omega t}(\mathbf{x}_\mathrm{m}(\omega t)\big) + \frac{1}{2}(A^2\Sigma_{yy} + B^2\Sigma_{xx} - 2AB\cos(\phi_d)\Sigma_{xy}), \\ &= \Sigma_{xx}\Sigma_{yy} - \Sigma_{xy}^2 + \frac{1}{4}(AB)^2\sin^2(\phi_d) + \frac{1}{2}(A^2\Sigma_{yy} + B^2\Sigma_{xx} - 2AB\cos(\phi_d)\Sigma_{xy}) \end{aligned} \tag{72}$$

Using these expressions, one can evaluate the squared difference of the two eigenvalues as follows:

$$\begin{aligned} (\lambda'_+ - \lambda'_-)^2 =& \varsigma_1^2 - 4\varsigma_2 \\ =& (\Sigma_{xx} - \Sigma_{yy})^2 + 4\Sigma_{xy}^2 + \frac{1}{4}(A^2 - B^2)^2 + (AB)^2\cos^2(\phi_d) \\ &+ (A^2 + B^2)(\Sigma_{xx} - \Sigma_{yy}) + 4AB\cos(\phi_d)\Sigma_{xy}. \end{aligned} \tag{73}$$

Finally, the leading-order asymptotic variance of the estimated tilt is:

$$\mathrm{Var}(\varepsilon) \approx \frac{\lambda'_+\lambda'_-}{N_\mathrm{tot}\,(\lambda'_+ - \lambda'_-)^2} = \frac{\varsigma_2}{N_\mathrm{tot}\,(\lambda'_+ - \lambda'_-)^2}, \tag{74}$$

where we insert the results in Eq. (72) and Eq. (73)

We now study some representative regimes under the assumption that the measurement covariance does not induce correlations between x and y, i.e. $\Sigma_{xy} = 0$.

1) Strong MW driving field, negligible beam convergence. In this regime, all the components of $\mathbf{\Sigma}$ are much smaller than A^2 and B^2, which represent the signal amplitudes associated with the mean deflection. Under this assumption, we find that

$$\lambda'_+\lambda'_- = \varsigma_2 \approx \frac{1}{4}(AB)^2\sin^2(\phi_d) + \frac{1}{2}(A^2\Sigma_{yy} + B^2\Sigma_{xx}),$$
$$(\lambda'_+ - \lambda'_-)^2 \approx \frac{1}{4}(A^4 + B^4) + \frac{1}{2}(AB)^2\cos(2\phi_d) + (A^2 + B^2)(\Sigma_{xx} - \Sigma_{yy}).$$

Then, the variance of the estimated tilting angle is approximately

$$\mathrm{Var}(\varepsilon) \approx \frac{1}{N_{\mathrm{tot}}}\left(\frac{A^2B^2\sin^2(\phi_d)}{A^4 + B^4 + 2A^2B^2\cos(2\phi_d)}\right). \tag{75}$$

The variance depends solely on the MW driving field amplitude (A and B) and the phase delay ϕ_d between components. For $\phi_d = 0$, the variance vanishes ($\mathrm{Var}(\varepsilon) \to 0$) because the data points lie along a perfectly aligned line, allowing the tilt angle to be determined with complete certainty. For $\phi_d = \pi/2$ and $A = B$, the variance diverges ($\mathrm{Var}(\varepsilon) \to \infty$), as the data points trace a circle. Finally, for $\phi_d = \pi/2$ and $A \gg B$, the variance follows

$$\mathrm{Var}(\varepsilon) \approx \frac{1}{N_{\mathrm{tot}}}\left(\frac{B^2}{A^2}\right).$$

In further examples, we assume $\phi_d = 0$, corresponding to the optimized experimental configuration.

2) Strong MW driving field, small beam convergence (parallel beam). We assume $\phi_d = 0$ and the elements of $\mathbf{\Sigma}$ are small compared to A^2 and B^2. Such a configuration best represents the experiments with the parallel electron beam in the main text. The resulting

variance is

$$\mathrm{Var}(\varepsilon) \approx \frac{1}{N_{\mathrm{tot}}} \left(\frac{2(A^2\Sigma_{yy} + B^2\Sigma_{xx})}{(A^2+B^2)^2} \right) \left(1 - 4\frac{\Sigma_{xx} - \Sigma_{yy}}{A^2+B^2} \right). \tag{76}$$

If the gaussian spread is isotropic, i.e. $\Sigma_{xx} = \Sigma_{yy}$, and the beam deflection $\alpha_{\mathrm{max}} = A$ for $A \gg B$, the variance simplifies to

$$\mathrm{Var}(\varepsilon) \approx \frac{1}{N_{\mathrm{tot}}} \left(\frac{2\Sigma_{xx}}{\alpha_{\mathrm{max}}^2} \right).$$

This result indicates that the variance is linearly dependent on the Gaussian spread of the electron beam and decreases quadratically with the deflection of the electron beam. Hence, precise angle estimation benefits from sufficiently large beam deflection.

In the experiment, we compute the pattern-endpoint deflection $\gamma_{\mathrm{max}} = \alpha_{\mathrm{max}}\varepsilon$ using the small-angle approximation. We assume that the value of α_{max} is known precisely (although, in practice, this is not strictly the case). Therefore, the variance propagates as

$$\mathrm{Var}(\gamma_{\mathrm{max}}) = \alpha_{\mathrm{max}}^2 \,\mathrm{Var}(\varepsilon) \approx \frac{2\Sigma_{xx}}{N_{\mathrm{tot}}}. \tag{77}$$

By contrast, the deflection precision of conventional techniques, such as the COM method, follows[S12,S13]

$$\mathrm{Var}(\mathrm{COM}) \approx \frac{\Sigma_{xx}}{N_{\mathrm{tot}}}.$$

Both expressions exhibit the same scaling with $1/N_{\mathrm{tot}}$ and the Gaussian width Σ_{xx}, indicating that the variance decreases inversely with the number of measurements and increases proportionally with the beam spread. Although the PCA-based approach reduces the nominal deflection sensitivity by a factor of 2 compared with the COM method, it offers several important advantages. By not relying on the absolute COM, the analysis is robust to drifts and jumps of the electron beam pattern. As long as the noise sources, such as electromagnetic fluctuations, do not induce a tilt, the tilt angle can still be determined precisely. Moreover, PCA enables detection of time-varying processes via phase-locked measurements,

whereas COM-based analysis requires direct time resolution of the signal with respect to the MW phase.

3) MW driving field deflection comparable with the beam convergence (focused STEM probe). In such a case, we must directly employ Eq. (74). However, if we assume that $A \gg B$, $\phi_d = 0$, and $\mathbf{\Sigma}$ on the same order as A, we obtain

$$\lambda'_+\lambda'_- = \varsigma_2 = \Sigma_{xx}\Sigma_{yy} + \frac{1}{2}A^2\Sigma_{yy}, \qquad (\lambda'_+ - \lambda'_-)^2 = (\Sigma_{xx} - \Sigma_{yy})^2 + \frac{1}{4}A^4 + A^2(\Sigma_{xx} - \Sigma_{yy}),$$

and therefore

$$\mathrm{Var}(\varepsilon) \approx \frac{1}{N_{\mathrm{tot}}}\left(\frac{2(2\Sigma_{xx}\Sigma_{yy} + A^2\Sigma_{yy})}{4(\Sigma_{xx} - \Sigma_{yy})^2 + A^4 + 4A^2(\Sigma_{xx} - \Sigma_{yy})} + \mathcal{O}\left(\frac{B^2}{A^2}\right)\right). \tag{78}$$

In the isotropic limit ($\Sigma_{xx} = \Sigma_{yy}$), for $\alpha_{\mathrm{max}} = A$ this simplifies to

$$\mathrm{Var}(\varepsilon) \approx \frac{1}{N_{\mathrm{tot}}}\frac{2\Sigma_{xx}(2\Sigma_{xx} + \alpha_{\mathrm{max}}^2)}{\alpha_{\mathrm{max}}^4}.$$

This expression explicitly shows that, when the MW-driven deflection satisfies $\alpha_{\mathrm{max}}^2 > \Sigma_{xx}$ (as in the experimental conditions of Fig. S5), the variance scales approximately as $\sim \Sigma_{xx}/\alpha_{\mathrm{max}}^2$. Conversely, for weak driving fields $\alpha_{\mathrm{max}}^2 < \Sigma_{xx}$, the variance scales as $\sim \Sigma_{xx}^2/\alpha_{\mathrm{max}}^4$, reflecting a loss of angular precision.

These theoretical results emphasize the capability of SPINEM to operate effectively even in a focused STEM probe configuration, as experimentally demonstrated in Fig. S5. Under strong MW excitation, resulting in a large α_{max} deflection, the tilt angle ε can be determined with high precision, and consequently the endpoint deflection γ_{max} can be established with a variance

$$\mathrm{Var}(\gamma_{\mathrm{max}}) = \alpha_{\mathrm{max}}^2\,\mathrm{Var}(\varepsilon) \approx \frac{1}{N_{\mathrm{tot}}}\frac{2\Sigma_{xx}(2\Sigma_{xx} + \alpha_{\mathrm{max}}^2)}{\alpha_{\mathrm{max}}^2}. \tag{79}$$

This quadratic dependence on α_{max} illustrates the improvement in deflection precision with

increasing MW drive strength and reduced beam spread, consistent with the experimentally observed behavior.

SPINEM Spin Sensitivity Estimate. For the final estimate of the spin sensitivity, N_{min}, of the SPINEM method, we follow Eq. 24 at $\omega t = 0$, corresponding to the obtainable signal at the endpoints of the deflection pattern:

$$\gamma_{\text{max}}(\mathbf{R}, \omega) = \mathcal{N} \frac{y^2 M_{y'} + 2xy M_{x'} - x^2 M_{y'}}{(x^2 + y^2)^2}. \tag{80}$$

Introducing polar coordinates, $x = R\cos p$ and $y = R\sin p$, this expression becomes

$$\gamma_{\text{max}}(\mathbf{R}, \omega) = \frac{\mathcal{N}}{R^2}\big(M_{x'} \sin 2p - M_{y'} \cos 2p\big). \tag{81}$$

The maximum deflection is obtained when the angular term reaches its extrema, yielding

$$\gamma_{\text{max}}(\mathbf{R}, \omega) = \frac{\mathcal{N}}{R^2}\sqrt{M_{x'}^2 + M_{y'}^2}. \tag{82}$$

This result shows that the maximum deflection scales as $1/R^2$ and is proportional to the magnitude of the transverse magnetization, $m = \sqrt{M_{x'}^2 + M_{y'}^2}$. For $T_1 = T_2$ and an optimally driven specimen, we have $m = \frac{1}{2}M_0$ and $\gamma_{\text{max}} = \frac{\mathcal{N} M_0}{2R^2}$. Using the result for the parallel beam (SI D.2 section 2), the SNR of the SPINEM method follows

$$\text{SNR} = \frac{\gamma_{\text{max}}}{3\sqrt{\text{Var}(\gamma_{\text{max}})}} = \frac{1}{3}\frac{\mathcal{N} M_0}{2R^2}\sqrt{\frac{N_{\text{tot}}}{2\sigma^2}}, \tag{83}$$

where $\text{Var}(\gamma_{\text{max}})$ is given in Eq. (77), $\sigma^2 = \Sigma_{xx} = \Sigma_{yy}$ is the variance of the Gaussian electron beam profile, and the factor 3 stems from an SNR convention. The spin sensitivity of SPINEM is given by:

$$N_{\text{min}} = \frac{N_{\text{s}} V}{\text{SNR}}\sqrt{T_{\text{int}}} = \frac{12\sqrt{2}\pi m_{\text{e}}^* v_{\text{e}}}{e\mu_0}\frac{K_{\text{B}} T}{\mu_{\text{B}}^2 B_0}\frac{\sigma R^2}{\sqrt{I_{\text{e}}/e}}, \tag{84}$$

where T_{int} is the total integration time for each datapoint and I_{e} is the electron beam current. Table S2 summarizes the scaling dependencies.

Table S2: Scaling of SPINEM spin sensitivity N_{min} with key parameters. A lower N_{min} corresponds to higher sensitivity.

**Parameter**	**Scaling of N_{min}**	**Origin**
Temperature T	$\propto T$	Spin polarization $\propto 1/T$
Bias field B_0	$\propto 1/B_0$	Zeeman splitting $\rightarrow$ spin polarization $\propto B_0$
Probe–spec. dist. R	$\propto R^2$	Stronger fields closer to the specimen
		Larger beam deflection $\delta_{\mathrm{max}} \propto \frac{1}{\mathbf{R}^2}$
Beam angular width σ	$\propto \sigma$	Wider spot $\rightarrow$ larger COM uncertainty $\propto \sigma$
El. current I_{e}	$\propto 1/\sqrt{I_{\mathrm{e}}}$	Larger information transfer
Electron speed v_e	$\propto v_e$	Slower electrons $\rightarrow$ longer interaction time
		and larger deflection angles

Several key insights emerge from comparing the sensitivity of SPINEM and conventional ESR. Both methods benefit from reduced temperature T[S14,S15] and larger bias magnetic field B_0, although SPINEM scales linearly in these parameters. In both cases, sensitivity increases significantly with a decrease in the distance between the sensing device (microcoil or free-space electron probe) and the specimen. This scaling is particularly advantageous for SPINEM, as the TEM can probe extremely close to the spin system, achieving a sub-100 pm spatial resolution.[S16–S18]

From the measurement presented in Fig. 2, we extract a spin sensitivity of $N_{\mathrm{min}} \approx 5.6 \times 10^{14}$ spins/$\sqrt{\mathrm{Hz}}$. This value was obtained using a subset of the data with a 5 s acquisition window for each frame. Although this subset exhibits a slightly lower SNR of approximately 24, it yields improved spin sensitivity because the variance relevant for N_{min} is less affected by slow environmental and instrumental drifts. At short integration times, the detection is primarily limited by electron shot noise, leading to the familiar $1/\sqrt{t}$ scaling of the SNR. However, for longer acquisitions, slow drifts of the electron beam, sample stage, or environmental conditions begin to dominate, causing the SNR to increase more slowly than predicted by shot-noise scaling. In our experiment, this transition from shot-noise-limited to drift-limited behavior occurs on timescales below 20 s. Consequently, using the full 20 s

dataset only marginally improves the SNR, and the corresponding spin sensitivity decreases to $N_{\mathrm{min}} \approx 1 \times 10^{15}$ spins/$\sqrt{\mathrm{Hz}}$.

For this reason, we report the optimized N_{min} obtained from the shorter 5 s acquisition, despite the lower SNR. Extrapolating this behavior, an acquisition time of 100 s would not yield the tenfold SNR improvement expected from shot-noise-limited detection, but only a modest increase. In future implementations, increasing the electron beam current (requiring the use of a camera with a larger dynamic range) could allow the same SNR to be achieved in shorter acquisition times, thereby operating closer to the shot-noise limit and mitigating drift-related limitations.

Based on the experimental parameters (T = 300 K, B_0 = 174.4 mT, R = 150 µm, I_{e} = 3.9 pA, and a beam angular width of σ = 470 nrad measured at the largest part of the electron beam pattern of measurement in Fig. 2), the theoretical estimate yields $N_{\mathrm{min}} = 4.2 \cdot 10^{13}$ spins/$\sqrt{\mathrm{Hz}}$. As detailed in SI B.1, a factor of 2.2 accounts for part of this difference between theory and experimental estimations. The remaining discrepancy between the experimental and theoretical sensitivity estimates can be attributed to non-ideal measurement conditions, including stray magnetic fields and other noise sources. A first step to resolving these issues would be the implementation of mu-metal shielding around the TEM, while additional gains could be achieved by employing high speed direct electron detectors[S19] and advanced TEM correctors. It should also be noted that the theoretical estimates rely on simplifying assumptions, such as ideal spin system driving and the magnetic dipole approximation.

While the experimental SPINEM sensitivity does not yet surpass that of conventional ESR spectroscopy, the potential is clear. Reducing the probe-specimen distance to $R = 5$ nm, increasing the beam current to $I_{\mathrm{e}} = 500$ pA with an angular width of 200 µrad (parameters readily achievable with LM-STEM probe), the theoretical sensitivity would be improved to $N_{\mathrm{min}} = 5.5 \cdot 10^{4}$ spins/$\sqrt{\mathrm{Hz}}$, well beyond the reach of state-of-the-art ESR. Ultimately, SPINEM holds the promise of achieving single-spin sensitivity at the atomic scale.[S5,S20]

References

(S1) Künstner, S.; McPeak, J. E.; Chu, A.; Kern, M.; Wick, M.; Dinse, K.-P.; Anders, J.; Naydenov, B.; Lips, K. Microwave field mapping for EPR-on-a-chip experiments. *Science Advances* **2024**, *10*, eado5467.

(S2) Koelsch, C. Syntheses with triarylvinylmagnesium bromides. α, γ-Bisdiphenylene-β-phenylallyl, a stable free radical. *Journal of the American Chemical Society* **1957**, *79*, 4439–4441.

(S3) Boero, G.; Gualco, G.; Lisowski, R.; Anders, J.; Suter, D.; Brugger, J. Room temperature strong coupling between a microwave oscillator and an ensemble of electron spins. *Journal of Magnetic Resonance* **2013**, *231*, 133–140.

(S4) Goncalves, F. J.; Paterson, G. W.; McGrouther, D.; Drysdale, T.; Togawa, Y.; Schmool, D. S.; Stamps, R. L. Probing microwave fields and enabling in-situ experiments in a transmission electron microscope. *Scientific Reports* **2017**, *7*, 1–6.

(S5) Haslinger, P.; Nimmrichter, S.; Rätzel, D. Spin resonance spectroscopy with an electron microscope. *Quantum Science and Technology* **2024**, *9*, 035051.

(S6) Wilson, C. B.; Edwards, D. T.; Clayton, J. A.; Han, S.; Sherwin, M. S. Dressed Rabi oscillation in a crystalline organic radical. *Physical Review Letters* **2020**, *124*, 047201.

(S7) Jaroš, A.; Toyfl, J.; Pupić, A.; Czasch, B.; Boero, G.; Bicket, I. C.; Haslinger, P. Electron spin resonance spectroscopy in a transmission electron microscope. *Ultramicroscopy* **2025**, 114224.

(S8) Hoult, D. I.; Richards, R. E. The signal-to-noise ratio of the nuclear magnetic resonance experiment. *Journal of Magnetic Resonance* **1976**, *24*, 71–85.

(S9) Boero, G.; Bouterfas, M.; Massin, C.; Vincent, F.; Besse, P. A.; Popovic, R. S.;

Schweiger, A. Electron-spin resonance probe based on a 100 μm planar microcoil. *Review of Scientific Instruments* **2003**, *74*, 4794–4798.

(S10) Anderson, T. W. *An Introduction to Multivariate Statistical Analysis*, 3rd ed.; Wiley-Interscience: Hoboken, NJ, 2003; Chapter 7: The Distribution of the Sample Covariance Matrix and the Sample Generalized Variance.

(S11) Ross, S. M. *A first course in probability*, tenth edition, global edition. ed.; Pearson: Harlow, United Kingdom, 2020 - 2020; Chapter 7: Properties of Expectation.

(S12) Ishikawa, R.; Morishita, S.; Tanigaki, T.; Shibata, N.; Ikuhara, Y. Spatial and phase resolution in electron microscopy. *Microscopy* **2023**, *72*, 78–96.

(S13) Pöllath, S.; Schwarzhuber, F.; Zweck, J. The differential phase contrast uncertainty relation: connection between electron dose and field resolution. *Ultramicroscopy* **2021**, *228*, 113342.

(S14) Rennich, E.; Sung, S. H.; Agarwal, N.; Gates, M.; Kerns, R.; Hovden, R.; Baggari, I. E. Ultracold cryogenic TEM with liquid helium and high stability. *Proceedings of the National Academy of Sciences* **2025**, *122*, e2509736122.

(S15) Gatan, Inc. Cooling & In Situ TEM Holders. `https://www.gatan.com/products/tem-specimen-holders/cooling-situ-holders`.

(S16) Krivanek, O. L.; Bleloch, A. L.; Dellby, N.; Lovejoy, T. C.; Shi, C.; Zhou, W. Improving the STEM spatial resolution limit. *Microscopy and Microanalysis* **2018**, *24*, 18–19.

(S17) Stroppa, D. G.; Zagonel, L. F.; Montoro, L. A.; Leite, E. R.; Ramirez, A. J. High-Resolution Scanning Transmission Electron Microscopy (HRSTEM) Techniques: High-Resolution Imaging and Spectroscopy Side by Side. *ChemPhysChem* **2012**, *13*, 437–443.

(S18) Nguyen, K. X.; Huang, J.; Karigerasi, M. H.; Kang, K.; Cahill, D. G.; Zuo, J.-M.; Schleife, A.; Shoemaker, D. P.; Huang, P. Y. Angstrom-scale imaging of magnetization in antiferromagnetic Fe2As via 4D-STEM. *Ultramicroscopy* **2023**, *247*, 113696.

(S19) Bolzonella, R.; Alozy, J.; Ballabriga, R.; van Beuzekom, M.; Biesuz, N. V.; Campbell, M.; Cardarelli, P.; Cavallini, V.; Coco, V.; Ramusino, A. C.; others Timing resolution performance of Timepix4 bump-bonded assemblies. *Journal of Instrumentation* **2024**, *19*, P07021.

(S20) Beltrán-Romero, S.; Gaida, M.; Haslinger, P.; Rätzel, D.; Nimmrichter, S. Quantum Metrology of Spin Sensing with Free Space Electrons. *arXiv preprint arXiv:2509.14982* **2025**,
